# Boosting Ethylene/Ethane Separation within Copper(I)‐Chelated Metal–Organic Frameworks through Tailor‐Made Aperture and Specific π‐Complexation

**DOI:** 10.1002/advs.201901918

**Published:** 2019-11-25

**Authors:** Ling Zhang, Libo Li, Enlai Hu, Ling Yang, Kai Shao, Lijia Yao, Ke Jiang, Yuanjing Cui, Yu Yang, Bin Li, Banglin Chen, Guodong Qian

**Affiliations:** ^1^ State Key Laboratory of Silicon Materials Cyrus Tang Center for Sensor Materials and Applications School of Materials Science and Engineering Zhejiang University Zheda Road #38 Hangzhou 310027 China; ^2^ Shanxi Key Laboratory of Gas Energy Efficient and Clean Utilization College of Chemistry and Chemical Engineering Taiyuan University of Technology Taiyuan 030024 Shanxi China; ^3^ Department of Chemistry University of Texas at San Antonio One UTSA Circle San Antonio TX 78249‐0698 USA

**Keywords:** copper(I) ions, ethylene purification, gas selectivity, porous materials, size‐sieving, π‐complexation

## Abstract

The development of new materials for separating ethylene (C_2_H_4_) from ethane (C_2_H_6_) by adsorption is of great importance in the petrochemical industry, but remains very challenging owing to their close molecular sizes and physical properties. Using isoreticular chemistry in metal–organic frameworks (MOFs) enables the precise design and construction of target materials with suitable aperture sizes and functional sites for gas separations. Herein, it is described that fine‐tuning of pore size and π‐complexation simultaneously in microporous copper(I)‐chelated MOFs can remarkably boost the C_2_H_4_/C_2_H_6_ adsorption selectivity. The judicious choice of organic linkers with a different number of carboxyl groups in the UiO‐66 framework not only allows the fine tuning of the pore size but also immobilizes copper(I) ions onto the framework. The tailor‐made adsorbent, Cu^I^@UiO‐66‐(COOH)_2_, thus possesses the optimal pore window size and chelated Cu(I) ions to form π‐complexation with C_2_H_4_ molecules. It can rapidly adsorb C_2_H_4_ driven by the strong π‐complexation interactions, while effectively reducing C_2_H_6_ uptake due to the selective size‐sieving. Therefore, this material exhibits an ultrahigh C_2_H_4_/C_2_H_6_ selectivity (80.8), outperforming most previously described benchmark materials. The exceptional separation performance of Cu^I^@UiO‐66‐(COOH)_2_ is validated by breakthrough experiments for 50/50 v/v C_2_H_4_/C_2_H_6_ mixtures under ambient conditions.

## Graphical abstract

Fine‐tuning of pore size and π‐complexation simultaneously in microporous copper(I)‐chelated metal–organic frameworks remarkably boosts the C_2_H_4_/C_2_H_6_ adsorption selectivity. The tailor‐made copper(I)‐chelated adsorbent, Cu^I^@UiO‐66‐(COOH)_2_, rapidly adsorbs C_2_H_4_ driven by the strong π‐complexation interactions, while effectively reducing the C_2_H_6_ uptake due to the size‐sieving.

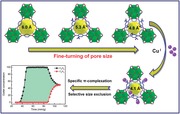

## 1 Introduction

Purification of olefins alone accounts for 0.3% of global energy consumption. Olefin/paraffin separation has been thus highlighted as one of seven most important chemical separations.^1^ Ethylene (C_2_H_4_), as the most important olefins, is the mainstay of petrochemical industry, with a global annual production of exceeding 170 million tonnes per year. “Polymer‐grade” specification of ethylene is required for the manufacture of polyethylene plastic. The industrial separation of ethylene from ethylene/ethane (C_2_H_4_/C_2_H_6_) mixtures highly relies on the repeated distillation–compression cycling at the temperature as low as −160 °C.^1^, ^2^ Such heat‐driven separation involving in the phase change of isolated fractions, is highly energy‐ and capital‐intensive. Finding energy‐efficient alternatives to distillation would widely lower global energy consumption, carbon emissions, and pollution. It is feasible in principle to separate C_2_H_4_/C_2_H_6_ mixtures based on porous solid materials via the energy‐efficient and environmentally friendly adsorption technology. In this context, development of suitable porous adsorbents for ethylene/ethane separation is of highly commercial significance.

A number of porous materials including zeolites,^3^ carbon molecular sieves,^4^ and alumina,^5^ have been explored for the separation of ethylene and ethane. However, the limits on deliberately designing the structure of such purely inorganic materials make them hardly meet the requirement of industrial implement. As an emerging class of microporous materials, metal–organic frameworks (MOFs) hold particular promise for the separation of light hydrocarbons,^6^, ^7^, ^8^, ^9^, ^10^, ^11^, ^12^, ^13^, ^14^, ^15^ because of their powerful reticular chemistry that enables them more readily tuning of their pore aperture and functionality.^16^, ^17^, ^18^, ^19^, ^20^, ^21^, ^22^ The most popular cases of C_2_H_4_/C_2_H_6_ separation at present have been mostly achieved by thermodynamic driven separation. One of the effective strategies is to immobilize the metal ions (such as Cu(I) and Ag(I)) on the pores to form the selective π‐complexation with ethylene, which has been well proved by porous materials.^23^, ^24^ Another analogous π‐complexation effect is typically contributed from open metal sites (OMSs) among MOFs.^25^ Such effect is capable of discriminating ethylene from ethane, and thus perform well for the separation of ethylene and ethane. Unfortunately, coadsorption of ethane commonly exists in these materials, originated from dynamic diffusion in oversize apertures and/or polarization of OMSs, thus delimiting the separation performance in most cases. On the other hand, C_2_H_4_/C_2_H_6_ separation can also be achieved by the controlled size‐sieving effect.^6^ However, considering their nearly identical sizes (only 0.028 nm difference in kinetic diameter), a few MOFs show the selective separation of C_2_H_4_/C_2_H_6_ based on this strategy with moderate high selectivities,^26^ and only one MOF (UTSA‐280) reported so far has shown the complete exclusion of ethane from C_2_H_4_/C_2_H_6_ mixtures with benchmark selectivity.^27^ Therefore, it still remains very challenging for MOF materials to separate ethylene from ethylene/ethane mixtures with a satisfied high selectivity based on a single separation mechanism. We speculate that if we combined the effect of π‐complexation and aperture sieving into a single MOF material, the resulting adsorbent may exhibit a superior separation performance for C_2_H_4_/C_2_H_6_. However, such synergistic effect has yet to be systematically studied within MOFs for this challenging separation.

**Figure 1:**
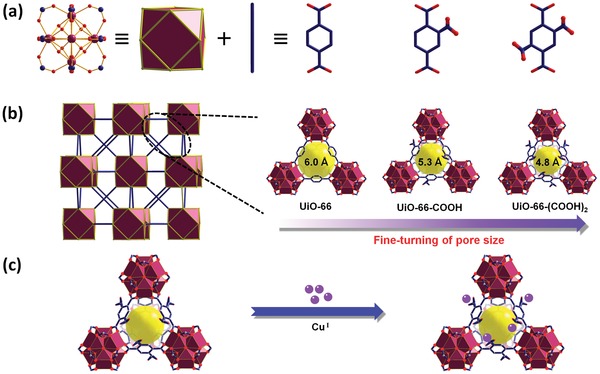
X‐ray single crystal structure of UiO‐66‐type MOFs, indicating that 2‐connected linkers bridge 12‐connected [Zr_6_(µ_3_‐OH)_8_(O_2_C—)_12_] molecular building blocks (MBBs) to form the 3D fcu‐topology frameworks. The pore window size can be systemically modulated via the judicious choice of organic linkers, and it can be further contracted after the configuration of copper(I) ions.

Herein, we selected the UiO‐66 family from the abundant MOF gene pool for the study of C_2_H_4_/C_2_H_6_ separation due to their typically predictable and readily designable pore size and functionality.^28^, ^29^, ^30^ In this work, we carefully single out two organic linkers with different number of carboxyl groups, namely benzene‐1,2,4‐tricarboxylic acid (1,2,4‐BTC) and benzene‐1,2,4,5‐tetracarboxylic acid (1,2,4,5‐BTEC), to construct two different fcu‐MOFs (termed as UiO‐66‐COOH and UiO‐66‐(COOH)_2_) by using isoreticular chemistry (**Figure** **1**). The judicious choice of organic linkers in the UiO‐66 framework allows us to not only finely tune the pore size but also immobilize copper(I) ions onto the framework. The tailor‐made copper(I)‐chelated adsorbent, Cu^I^@UiO‐66‐(COOH)_2_, thus possesses the optimal pore window size and specific π‐complexation for C_2_H_4_/C_2_H_6_ separation. Gas sorption studies show that Cu^Ι^@UiO‐66‐(COOH)_2_ can rapidly capture ethylene via the strong π‐complexation affinity, while effectively reduce ethane adsorption due to its size‐sieving effect. As a result, its ideal adsorbed solution theory (IAST) selectivity for 50/50 C_2_H_4_/C_2_H_6_ mixtures at ambient conditions can reach up to 80.8, only lower than the benchmark UTSA‐280^27^ but outdistancing all the other previously top‐performing materials, such as Zeolite 13X (13.4),^31^ NaETS‐10 (14.7),^32^ NOTT‐300 (48.7),^33^ Co‐gallate (52)^27^ and FeMOF‐74 (13.5),^34^ PAF‐SO_3_Ag (26.9).^24^ Its exceptional separation performance was further validated by the breakthrough experiments on 50/50 v/v C_2_H_4_/C_2_H_6_ mixtures under ambient conditions.

## 2 Results and Discussion

**Figure 2:**
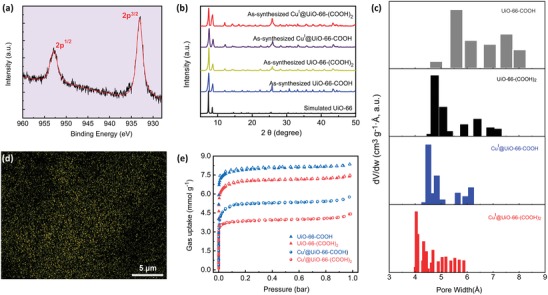
a) XPS for Cu(I) sites of Cu^Ι^@UiO‐66‐(COOH)_2_ after the surface etching; b) The PXRD patterns for the synthesized UiO‐66 series MOF materials along with the simulated XRD pattern of UiO‐66 (black) derived from the simulated crystal structure; c) The pore size distribution of UiO‐66 series MOF materials; d) Element maps for Cuprum (Cu) of Cu^Ι^@UiO‐66‐(COOH)_2_; e) N_2_ sorption isotherms at 77 K of UiO‐66 series MOF materials.

Encouraged by the successful construction of isoreticular UiO‐66 with deliberately fine‐tuned apertures,^30^ we elected to explore the promise of this fcu‐MOF platform for the adsorptive separation of ethylene form ethane. As shown in Figure 1, MOF materials with fcu topology hold a unique desired feature that the entrance into the inner pores of the frameworks is only via the ligand‐delimited triangular windows. Construction of isoreticular fcu‐MOF with relatively bulkier linkers will permit the anticipated contraction of pore apertures, and subsequently realize the efficient size‐sieving effect. With this in mind, we selected 2‐connected linkers with different number of carboxyl groups (1,2,4‐BTC and 1,2,4,5‐BTEC) to construct two different fcu‐MOFs (UiO‐66‐COOH and UiO‐66‐(COOH)_2_) by using isoreticular chemistry. Both of the synthesized MOFs have the prospective fcu topology as determined by the PXRD analyses (**Figure** **2**b), in which the patterns of both UiO‐66‐(COOH)_2_ and UiO‐66‐COOH match well with the theoretical ones of UiO‐66 derived from the crystal structure. Topological analysis of the UiO‐66 series indicates that the carbon atoms from the coordinated carboxylates act the extension points of network, bridging the 2‐connected linkers and the 12‐connected [Zr_6_(µ_3_‐OH)_8_(O_2_C−)_12_] molecular building blocks (MBBs) to produce the 3D framework (Figure 1a,b). The window size of UiO‐66 was found to be around 6.0 Å. Obviously, both ethylene and ethane molecules can readily diffuse into the internal pores of UiO‐66. When using the bulkier linker of 1,2,4‐BTC instead of 1,4‐BDC, the resulting UiO‐66‐COOH shows a contracted pore window size of 5.3 Å. Further reduction in window size can be fulfilled by using 1,2,4,5‐BTEC to construct the framework. The corresponding size is reduced to be 4.8 Å for UiO‐66‐(COOH)_2_. In principle, such pore aperture is still much larger than the sizes of both C_2_H_4_ (4.1 Å) and C_2_H_6_ (4.4 Å), and cannot provide the obvious size‐sieving effect for C_2_H_4_/C_2_H_6_ separation. However, these functionalized linkers with free carboxyl groups provide us a platform to immobilize some targeted metal sites and thus to further reduce the pore window size (Figure 1c).

It has been well documented that the incorporated Cu(Ι) and Ag(Ι) ions into porous materials can form the specific π‐complexation with alkenyl units of olefin molecules to fulfill high separation performance.^23^, ^24^ Owing to the existing free carboxyl groups in Cu^Ι^@UiO‐66‐COOH and Cu^Ι^@UiO‐66‐(COOH)_2_, it is reasonable to speculate that the incorporation of such transition metals into these MOFs may be highly effective to discriminate ethylene and ethane significantly. On the other hand, this process can also reduce their pore window sizes and thus make them provide more efficient size‐sieving effect to further improve the separation performance. Therefore, we herein introduced the Cu(I) ions into the aforementioned fcu‐MOF materials by the coordination with bare carboxyl groups to form two Cu(I) immobilized materials, namely Cu^Ι^@UiO‐66‐COOH and Cu^Ι^@UiO‐66‐(COOH)_2_. First, we confirmed the presence of Cu(I) ions in these metalated materials by X‐ray photoelectron spectroscopy (XPS) analyses (Figure 2a; Figure S5, Supporting Information). There exists a characteristic signal from copper(I) at binding energies of 952.9 and 933.1 eV, corresponding to the peaks of Cu 2p^1/2^ and 2p^3/2^, respectively. Then, their phase purity of bulk materials was determined by the PXRD analysis (Figure 2b and S1, Supporting Information). FE‐SEM was performed to characterize the morphology of MOF materials (Figure S2, Supporting Information), all of which feature the homogeneous particles with a size of 100–200 nm. Apparently, the immobilization process shows no significant effect on the morphology of these robust MOFs. Fourier infrared spectrum analysis (FTIR) indicated that a strong band at 1715.7 cm^−1^ can be clearly observed in UiO‐66‐COOH and UiO‐66‐(COOH)_2_, mainly attributed to the C=O stretching vibration of uncoordinated ‐COOH groups (Figure S3, Supporting Information). After the metalation of two MOFs, this peak almost disappeared in Cu^Ι^@UiO‐66‐(COOH)_2_ and Cu^Ι^@UiO‐66‐COOH, further confirming the success of chelating the Cu(I) ions into carboxyl groups. The ICP‐MS (Table S1, Supporting Information) and energy‐dispersive X‐ray (EDS) analyses (Figure 2d; Figure S6, Supporting Information) comprehensively determined that there are ≈47% and ≈52% uncoordinated –COOH groups transformed into –COOCu for Cu^Ι^@UiO‐66‐COOH and Cu^Ι^@UiO‐66‐(COOH)_2_, respectively.

Permanent porosity studies of these four UiO‐66‐type MOFs were performed by nitrogen (N_2_) sorption at 77 K, and all isothermals show typically reversible type‐I isothermals (Figure 2c,e). The Brunauer–Emmett–Teller (BET) surface area was estimated to be 712.8 and 622.3 m^2^ g^−1^ for UiO‐66‐COOH and UiO‐66‐(COOH)_2_ respectively, notably lower than that of the parent UiO‐66 (1110 m^2^ g^−1^) due to the bulkier linkers.^35^ With the copper ions chelated into the MOFs, the porosity can be further reduced and the BET surface area of Cu^Ι^@UiO‐66‐COOH and Cu^Ι^@UiO‐66‐(COOH)_2_ decreased to be 437.7 and 319.7 m^2^ g^−1^, respectively. The variation of pore sizes among these MOFs, determined by Horvath–Kawazoe method, corresponds well to the results of the porosities. As shown in Figure 2c, the pore size is also gradually reduced from 5.6 Å in UiO‐66‐COOH to 4.1 Å in Cu^Ι^@UiO‐66‐(COOH)_2_, with the increased number of functional carboxyl groups and the chelation of copper ions. These experimental results are consistent well with the aforementioned pore sizes calculated from the crystal structures. The optimal aperture (4.1 Å) of Cu^Ι^@UiO‐66‐(COOH)_2_ just falls in the range of the kinetic diameter of C_2_H_4_ (4.1 Å) and C_2_H_6_ (4.4 Å), which may provide an efficient size‐sieving effect on C_2_H_4_/C_2_H_6_ separation.

**Figure 3:**
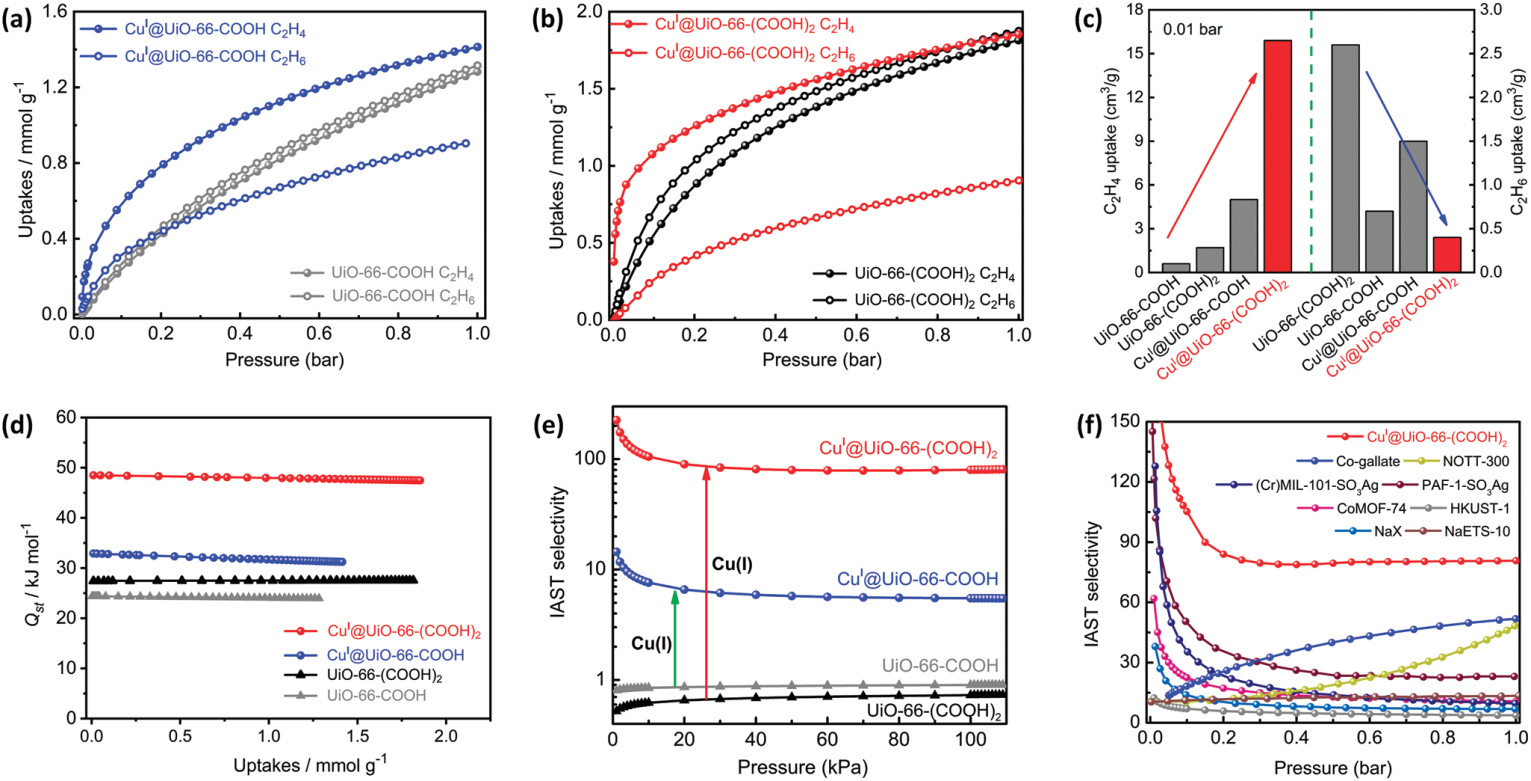
a) Single‐component adsorption isotherms for C_2_H_4_ and C_2_H_6_ of UiO‐66‐COOH and Cu^Ι^@UiO‐66‐COOH at 298 K; b) Single‐component adsorption isotherms for C_2_H_4_ and C_2_H_6_ of UiO‐66‐(COOH)_2_ and Cu^Ι^@UiO‐66‐(COOH)_2_ at 298 K; c) Experimental C_2_H_4_ and C_2_H_6_ adsorption uptake of UiO‐66 series MOF materials at 0.01 bar; d) The isosteric heat (*Q*_st_) of C_2_H_4_ adsorption in the UiO‐66‐type MOFs; e) IAST calculations of activated UiO‐66‐type MOFs for the C_2_H_4_/C_2_H_6_ separation at 298 K; f) IAST calculations of representative materials explored for C_2_H_4_/C_2_H_6_ separation at room temperature.

Single‐component adsorption isotherms of C_2_H_4_ and C_2_H_6_ including low‐pressure adsorption data for all four UiO‐66‐type MOFs were collected and shown in **Figure** **3**a,b; Figures S9 and S10 (Supporting Information). Evidently, both of UiO‐66‐COOH and UiO‐66‐(COOH)_2_ without Cu(I) ions show the very similar adsorption capacities toward C_2_H_4_ and C_2_H_6_, indicating that neither of them can discriminate the two gases due to their oversize aperture and the absence of specific recognition sites. Conversely, both copper‐chelated materials show distinct ethylene‐selective sorption behaviors. Especially, Cu^Ι^@UiO‐66‐(COOH)_2_ adsorbs C_2_H_4_ rapidly at low‐pressure region, with an appreciable uptake capacity of 1.86 mmol g^−1^ at 298 K and 1.0 bar. This C_2_H_4_ uptake at 1.0 bar approaches the stoichiometric quantity (2.14 mmol g^−1^) expected if one gas molecule is adsorbed per Cu(I) sites, indicating that the Cu(I) ions mainly account for its C_2_H_4_ uptake. Besides the increased C_2_H_4_ uptake, Cu^Ι^@UiO‐66‐COOH and Cu^Ι^@UiO‐66‐(COOH)_2_ also prove the notably reduced ethane uptake, indicating an efficient size‐selective effect. Cu^Ι^@UiO‐66‐COOH reduces ≈35.6% uptake for C_2_H_6_ at ambient conditions compared with UiO‐66‐COOH, and about 51.3%‐decreased uptake of C_2_H_6_ occurs on Cu^Ι^@UiO‐66‐(COOH)_2_ at the same conditions. As illustrated in Figure 3c, when the pore size of fcu MOFs gradually decreases from UiO‐66‐COOH to Cu^Ι^@UiO‐66‐(COOH)_2_, the uptake capacity of C_2_H_6_ at 0.01 bar becomes fewer and fewer. In contrast, the C_2_H_4_ uptake at 0.01 bar increases in the order of UiO‐66‐COOH < UiO‐66‐(COOH)_2_ < Cu^Ι^@UiO‐66‐COOH <Cu^Ι^@UiO‐66‐(COOH)_2_. Thus, the tailor‐made Cu^Ι^@UiO‐66‐(COOH)_2_ features the highest C_2_H_4_ adsorption (0.71 mmol g^−1^) but the lowest *C_2_*H_6_ adsorption (0.02 mmol g^−1^), thus offering an ultrahigh C_2_H_4_/C_2_H_6_ uptake ratio of 35.5 at 0.01 bar and 298 K. In comparison to other promising MOFs, both the C_2_H_4_ capture capacity and the low C_2_H_6_ uptake at low pressure are also very significant (Figures S11 and S12, Supporting Information). These results clearly indicate that the immobilization of Cu(I) ions into Cu^Ι^@UiO‐66‐(COOH)_2_ can efficiently improve the C_2_H_4_ capture capacity via the strong π‐complexation interactions, while the contracted aperture size provides the size‐sieving effect to reduce C_2_H_6_ uptake simultaneously, thus affording the excellent C_2_H_4_/C_2_H_6_ separation.

Such C_2_H_4_ adsorption behavior of four fcu‐MOFs can be explained by the isosteric heat of adsorption (*Q*_st_), calculated by adsorption isotherms at different temperatures. As shown in Figure 3d and Figure S15 (Supporting Information), the *Q*_st_ results are in good agreement with the experimental C_2_H_4_ uptakes. Higher *Q*_st_ values of C_2_H_4_ were found in both Cu(I)‐chelated MOFs, thus validating that Cu(I) ions indeed enhance the binding affinity of C_2_H_4_. The *Q*_st_ value (48.5 kJ mol^−1^) for C_2_H_4_ at close to zero loading in Cu^Ι^@UiO‐66‐(COOH)_2_ is the highest, followed by Cu^Ι^@UiO‐66‐COOH (32.9 kJ mol^−1^), UiO‐66‐(COOH)_2_ (27.4 kJ mol^−1^), and UiO‐66‐COOH (24.3 kJ mol^−1^). This C_2_H_4_ *Q*_st_ value of Cu^Ι^@UiO‐66‐(COOH)_2_ is even comparable to that of M_2_(dobdc)^25^ with high density of OMSs, implying the strong affinity between Cu^Ι^@UiO‐66‐(COOH)_2_ and C_2_H_4_. We speculate that such strong C_2_H_4_ affinity is mainly attributed to the synergistic effect of the specific π‐complexation interactions and the smaller pores in Cu^Ι^@UiO‐66‐(COOH)_2_. Instead, the incorporation of Cu(I) ions shows a negative effect on the affinity of ethane, in which the *Q*_st_ values of C_2_H_6_ for Cu^Ι^@UiO‐66‐COOH and Cu^Ι^@UiO‐66‐(COOH)_2_ are even lower than the unmetalized MOFs. It is worth to note that the *Q*_st_ value of C_2_H_4_ in Cu^Ι^@UiO‐66‐(COOH)_2_ is much lower than that of most Ag^Ι^‐chelated porous adsorbents, including PAF‐1‐SO_3_Ag (106 kJ mol^−1^)^24^ and (Cr)‐MIL‐101‐SO_3_Ag (120^36^/63^37^ kJ mol^−1^), affording a relatively low regeneration energy among these kinds of materials.

The C_2_H_4_/C_2_H_6_ adsorption selectivity (*S*_ads_) of these UiO‐66 materials was calculated by IAST based on the measured sorption isotherms. Figure 3e shows the data obtained at 298 K. Due to the very similar C_2_H_4_ and C_2_H_6_ adsorption isotherms, the *S*_ads_ value of UiO‐66‐COOH is as low as 0.9. Unlike UiO‐66‐COOH, the Cu(I)‐chelated Cu^Ι^@UiO‐66‐COOH shows a significantly improved *S*_ads_ value up to 14.5. With the tailor‐made apertures and Cu(I) ions, we found that Cu^Ι^@UiO‐66‐(COOH)_2_ exhibits an ultrahigh *S*_ads_ up to 225.7 at 0.01 bar and decreases down to 80.8 at 1.0 bar, which is orders of magnitude higher than all the other UiO‐66 materials. We note that this *S*_ads_ value of Cu^Ι^@UiO‐66‐(COOH)_2_ is only next to UTSA‐280,^27^ outperforming all the other benchmark materials including Zeolite 13X (13.4),^31^ NaETS‐10 (14.7),^32^ Co‐gallate (52),^27^ NOTT‐300 (48.7)^33^ (Figure 3f). In addition, it is also significantly higher than Ag(I)‐chelated adsorbents like PAF‐1‐SO_3_Ag (27)^24^ and (Cr)‐MIL‐101‐SO_3_Ag (16^36^/9.6^37^). Considering its lower C_2_H_4_ *Q*_st_ compared with these Ag(I)‐chelated adsorbents, the size‐sieving effect on Cu^Ι^@UiO‐66‐(COOH)_2_ also contributes significantly to its exceptional selectivity apart from the π‐complexation interactions. Moreover, the sieving effect of C_2_H_4_/C_2_H_6_ separation in Cu^Ι^@UiO‐66‐(COOH)_2_ can be strengthened at low temperature of 273 K, wherein the corresponding *S*_ads_ value can be further enhanced to 110 at 1 bar (Figure S20, Supporting Information).

**Figure 4:**
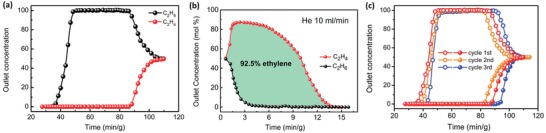
Multicomponent column breakthrough results for Cu^Ι^@UiO‐66‐(COOH)_2_ at 298 K. a) The breakthrough curves of Cu^Ι^@UiO‐66‐(COOH)_2_ for the C_2_H_4_/C_2_H_6_ (50/50, v/v) separation; b) The desorption curves of Cu^Ι^@UiO‐66‐(COOH)_2_ under 10.0 mL min^−1^ sweeping He gas at 413 K; c) Multiple cycles of breakthrough tests of Cu^Ι^@UiO‐66‐(COOH)_2_ for the C_2_H_4_/ C_2_H_6_ (50/50, v/v) separation.

The strong C_2_H_4_ capture capacity and ultrahigh IAST selectivity of Cu^Ι^@UiO‐66‐(COOH)_2_ prompted us to perform the breakthrough experiments in order to evaluate its actual separation efficiency. Such experiments were conducted in a packed column filled with the activated Cu^Ι^@UiO‐66‐(COOH)_2_ powder, under 1.0 mL min^−1^ feed gas of equimolar C_2_H_4_/C_2_H_6_ mixture at 298 K (**Figure** **4**; Figure S27, Supporting Information). As shown in Figure 4a, efficient separation of C_2_H_4_ from 50/50 C_2_H_4_/C_2_H_6_ mixtures can be successfully fulfilled by using the activated Cu^Ι^@UiO‐66‐(COOH)_2_. C_2_H_6_ gas was first eluted through the separation bed, while no C_2_H_4_ was detected until about 86 min. The adsorbed amount of C_2_H_4_, enriched from the equimolar C_2_H_4_/C_2_H_6_ mixtures, was calculated to be 1.92 mol per kg for a given cycle. Subsequently, the captured C_2_H_4_ gas in the column can then be recovered with high purity during the regeneration desorption step, which was carried out by the sweeping He gas (10 mL min^−1^) at 413 K. As shown in Figure 4b, the regeneration of Cu^Ι^@UiO‐66‐(COOH)_2_ revealed that the adsorbed gas can be almost completely recovered within 15 min and no detectable C_2_H_4_ and C_2_H_6_ were found after 15 min, notably faster than that of Ag‐doped adsorbent (typically thousands of minutes).^24^ Moreover, high pure (92.5%) ethylene can be obtained during one cycle of the adsorption–desorption procedures. Multiple cycling column breakthrough tests under the same conditions showed that the breakthrough times of Cu^Ι^@UiO‐66‐(COOH)_2_ for both C_2_H_4_ and C_2_H_6_ remains almost unchanged within three continuous cycles, confirming its good recyclability for C_2_H_4_/C_2_H_6_ separation (Figure 4c). Furthermore, Cu^Ι^@UiO‐66‐(COOH)_2_ was proved to be insensitive to moisture, since its C_2_H_4_ adsorption is not affected after soaking it into oxygen‐free water (Figure S29, Supporting Information).

## 3 Conclusions

Herein, we precisely designed and constructed two copper(I)‐chelated metal–organic frameworks by using isoreticular chemistry, for the very challenging ethylene/ethane separation. The tailor‐made adsorbent, Cu^I^@UiO‐66‐(COOH)_2_, possesses the optimal pore window size and specific π‐complexation. Our foregoing results indicated that this material can rapidly adsorb ethylene driven by the strong affinity of π‐complexation, while notably lessen ethane uptake due to its size‐sieving effect. This rare synergistic effect of the specific π‐complexation interactions and efficient size‐sieving effect in Cu^I^@UiO‐66‐(COOH)_2_ thus led to an ultrahigh IAST selectivity of 80.8 at ambient conditions for 50/50 C_2_H_4_/C_2_H_6_ mixture, outdistancing most of previously benchmark porous materials reported. The exceptional separation performance was further confirmed by the detailed breakthrough experiments on 50/50 v/v C_2_H_4_/C_2_H_6_ mixtures. In light of its attractive design strategy and ultrahigh selectivity, we believe that Cu^Ι^@UiO‐66‐(COOH)_2_ is placed among the best‐performing porous materials for the challenging C_2_H_4_/C_2_H_6_ separation, and this work may provide some guidance to develop new porous materials for boosting olefin/paraffin separation performance.

## 4 Experimental Section

*Materials and Methods*: All reagents and solvents including the organic ligands, 1,2,4‐BTC and 1,2,4,5‐BTEC, used to construct the UiO‐66‐type MOFs, were commercially available and used without further purification. Powder X‐ray diffraction (PXRD) patterns were collected in the 2θ = 5°–50° range on an X'Pert PRO diffractometer with Cu Kα (λ = 1.542 Å) radiation at room temperature. Inductively coupled plasma‐mass spectrometry (ICP‐MS) was performed on a Thermo Scientific XSERIES 2 ICP‐MS system. Fourier transform infrared (FT‐IR) spectrum was recorded on Thermo Fisher Nicolet iS10 spectrometer using KBr pallets in the 500–4000 cm^−1^ range. The morphology was investigated using a field‐emission scanning electron microscopy (FE‐SEM, Hitachi S4800). All gas sorption isotherms of UiO‐66‐type MOFs were obtained from the Micromeritics ASAP 2020 surface area analyzer and pore size analyzer. An ice‐water bath and water bath were used for C_2_H_4_ and C_2_H_6_ gases adsorption isotherms at 273 and 298 K, respectively.

*Preparation of Cu^I^@UiO‐66‐(COOH)_2_ and Cu^I^@UiO‐66‐COOH*: The activated UiO‐66‐(COOH)_2_ (≈200 mg, 0.09 mmol) and cuprous chloride (CuCl, about 108 mg, 1.08 mmol) were dispersed in acetonitrile (3 mL) in a 20 mL Teflon‐capped vial which was tightly wrapped with polytetrafluoroethylene sealing tape. The foregoing procedures were carefully operated in a glovebox with positive N_2_ pressure. Then, the vials were heated in an oven at 80 °C for two weeks to afford the resultant color‐changed powder Cu^I^@UiO‐66‐(COOH)_2_. The preparation of Cu^I^@UiO‐66‐COOH was referred to the preparation of Cu^I^@UiO‐66‐(COOH)_2_, just the replace of the amount of cuprous chloride (≈62 mg, 0.62 mmol) and the activated UiO‐66‐COOH (≈200 mg, 0.10 mmol).

*Activation of Cu^I^@UiO‐66‐(COOH)_2_ and Cu^I^@UiO‐66‐COOH*: Before the gas sorption measurements, the obtained powder solids were directly solvent‐exchanged by methanol for at least ten times in a glovebox with positive N_2_ pressure. After solvent‐exchange, the powder materials were carefully transferred into in the adsorption tube, then were evaluated from the Micromeritics ASAP 2020 surface area analyzer at 373 K for 12 h and 413 K for another 12 h to yield the activated Cu^I^@UiO‐66‐COOH and Cu^I^@ UiO‐66‐(COOH)_2._

## Conflict of Interest

The authors declare no conflict of interest.

## Supporting information

Supporting Information
