# Supplementary material for: Boosting Ethylene/Ethane Separation within Copper(I)‐Chelated Metal–Organic Frameworks through Tailor‐Made Aperture and Specific π‐Complexation
Source: Adv Sci (Weinh). 2019 Nov 25;7(2):1901918. doi: 10.1002/advs.201901918 (PMC6974952; doi:10.1002/advs.201901918)
Supplement: Supplementary file 1 — Supporting Information [file ADVS-7-1901918-s001.pdf]

Copyright WILEY-VCH Verlag GmbH & Co. KGaA, 69469 Weinheim, Germany, 2019.

ADVANCED SCIENCE
Open Access

Supporting Information

for *Adv. Sci.,* DOI: 10.1002/advs.201901918

Boosting Ethylene/Ethane Separation within Copper(I)-Chelated Metal–Organic Frameworks through Tailor-Made Aperture and Specific π-Complexation

Ling Zhang, Libo Li, Enlai Hu, Ling Yang, Kai Shao, Lijia Yao, Ke Jiang, Yuanjing Cui, Yu Yang, Bin Li, Banglin Chen,* and Guodong Qian**

Supporting Information

Boosting Ethylene/Ethane Separation within Copper(I)-Chelated Metal-Organic Frameworks Through Tailor-Made Aperture and Specific π-Complexation

Ling Zhang,[a] Libo Li, [c]Enlai Hu, [a] Ling Yang,[c] Kai Shao, [a] Lijia Yao, [a] Ke Jiang,[a] Yuanjing Cui,[a] Yu Yang,[a] Bin Li[a], Banglin Chen*[b] and Guodong Qian*[a]*

[a] State Key Laboratory of Silicon Materials, Cyrus Tang Center for Sensor Materials and Applications, School of Materials Science and Engineering, Zhejiang University, Hangzhou 310027, China

[b] Department of Chemistry, University of Texas at San Antonio, One UTSA Circle, San Antonio, Texas 78249-0698, United States.

[c] Shanxi Key Laboratory of Gas Energy Efficient and Clean Utilization, College of Chemistry and Chemical Engineering, Taiyuan University of Technology, Taiyuan, 030024, Shanxi, China.

Corresponding Author: bin.li@zju.edu.cn; banglin.chen@utsa.edu; gdqian@zju.edu.cn.

Table of Contents

Synthesis of UiO-66-COOH and UiO-66-$(COOH)_2$	S3
Ideal Adsorbed Solution Theory (IAST)	S3
Virial Graph Analysis for Q_{st}	S4
Breakthrough Tests	S5
The PXRD patterns	S6
SEM images	S7
FT-IR spectra	S8
Element maps	S9
XPS analyses	S10
Element analyses and ICP-MS	S11
Thermogravimetric analysis (TGA) curves	S12
The ESR spectra	S13
Single-component adsorption isotherms at 273 K	S14
Single-component adsorption isotherms at 298 K	S15
Comparison of C_2H_4 and C_2H_6 uptake capacity	S16
The fitting by the virial equation	S17
The isosteric heat (Q_{st}) of C_2H_6	S20
Langmuir-Freundlich parameter fits	S21
IAST calculation of C_2H_4/C_2H_6 separation	S25
IAST calculated C_2H_4 and C_2H_6 uptake	S30
Comparison of the selectivity of C_2H_4/C_2H_6	S34
The procedures for the breakthrough test	S35
Cycles of C_2H_4 adsorption	S38
The water stability of C_2H_4 adsorption	S39
Reference	S40

Experimental and Analysis Section

Synthesis of UiO-66-COOH and UiO-66-(COOH)$_2$

Both UiO-66-COOH and UiO-66-(COOH)$_2$ were synthesized by the reflux reaction according to the previously explored methods with a slight modification. Benzene-1,2,4,5-tetracarboxylic acid (1,2,4-BTeC) (0.85 g, 3.33 mmol) and zirconium tetrachloride ($ZrCl_4$) (0.81 g, 3.47 mmol) were dispersed in distilled water (20 mL) in a round-bottom flask, particularly adding acetic acid (13.3 mL) as a reaction modulator, then stirring for about 5 minutes until the mixture turned into clarified. The resulting reaction mixture was heated at 100 ℃ under reflux for 24 h, thus to yield a white powder UiO-66-(COOH)$_2$. The synthesis of UiO-66-COOH was referred to the synthesis of UiO-66-(COOH)$_2$, in which just the ligand of 1,2,4-BTeC (0.81 g, 3.47 mmol) was replaced by benzene-1,2,4-tricarboxylic acid (1,2,4-BTC) (0.70 g, 3.33 mmol).

Activation of UiO-66-COOH and UiO-66-(COOH)$_2$

Before the gas sorption measurements, the fresh powder solids were isolated by centrifugation for 8 min at 12000 rpm, then extensively washed with distilled water for at least five times, followed by solvent-exchanged with methanol over 10 times for about 5 days. After solvent-exchange, the powder materials were evaluated from the Micromeritics ASAP 2020 surface area analyzer at 373 K for 24 h to yield the activated UiO-66-COOH and UiO-66-(COOH)$_{2.}$

Ideal Adsorbed Solution Theory (IAST)

The selectivity of preferential adsorption of component 1 over component 2 in a mixture containing 1 and 2, can be formally defined as

$$S_{ads} = \frac{q1/q2}{p1/p2}$$

In above equation, q1 and q2 are the absolute component loadings of the adsorbed phase in the mixture. These component loadings are also termed the uptake capacity. We calculated the values of q1 and q2 using the Ideal Adsorbed Solution Theory (IAST) of Myers and Prausnitz.

Virial Graph Analysis: Estimation of the isosteric heats of gas adsorption (Q_{st}).

A virial-type expression of comprising the temperature-independent parameters a_i and b_j was employed to calculate the enthalpies of adsorption for C_2H_4 and C_2H_6 (at 273 K and 298 K) on **1**. In each case, the data were fitted use equation:

$$\ln P = \ln N + 1/T\sum_{i=0}^{m} a_i N_i + \sum_{j=0}^{n} b_j N_j \qquad (1)$$

Here, P is the pressure expressed in Pa, N is the amount absorbed in mmol g^{-1}, T is the temperature in K, a_i and b_j are virial coefficients, and m, n represent the number of coefficients required to adequately describe the isotherms (m and n were gradually increased till the contribution of extra added a and b coefficients was deemed to be statistically insignificant towards the overall fit. And the average value of the squared deviations from the experimental values was minimized). The values of the virial coefficients a_0 through a_m were then used to calculate the isosteric heat of absorption using the following expression:

$$Q_{st} = -R\sum_{i=0}^{m} a_i N_i \qquad (2)$$

Q_{st} is the coverage-dependent isosteric heat of adsorption and R is the universal gas constant. The heat enthalpies of C_2H_4 and C_2H_6 sorption for UiO-66-type MOFs in this manuscript are determined by using the sorption data measured in the pressure range from 0-1 bar (at 273 K and 298 K).

Breakthrough Tests.

Before the breakthrough experiments, the obtained powder Cu^I@ UiO-66-$(COOH)_2$ were directly solvent-exchanged by acetone for at least 10 times in a glovebox with positive N_2 pressure. After solvent-exchange, the powder materials were carefully transferred into in the adsorption tube, then were evaluated from the Micromeritics ASAP 2020 surface area analyzer at 373 K for 24 h and 413 K for another 24 h to yield the activated Cu^I@ UiO-66-$(COOH)_2$. The breakthrough experiments were conducted in a packed column (4.6 mm × 100 mm) filled with the activated Cu^I@UiO-66-$(COOH)_2$ samples. The column was first purged with He (15 mL min^{-1}) for 12 h at 298 K with packed sample. Then, the breakthrough tests were conducted under a mixed (C_2H_4/ C_2H_6, 50/50, v/v) dynamic feed gas with a flow of 1.0 mL min^{-1} at 298 K. The desorption tests of Cu^I@UiO-66-$(COOH)_2$ were conducted under a sweeping He gas with a flow of 10.0 mL min^{-1} at 298 K. After the breakthrough tests, Cu^I@UiO-66-$(COOH)_2$ can be readily regenerated under a sweeping He gas with a flow of 10.0 mL min^{-1} at 413 K for 2 h. All the outlet gases from the column were monitored using gas chromatography (GC-2010 plus, SHIMADZU) with a flame ionization detector (FID).

Supporting Figures and Tables

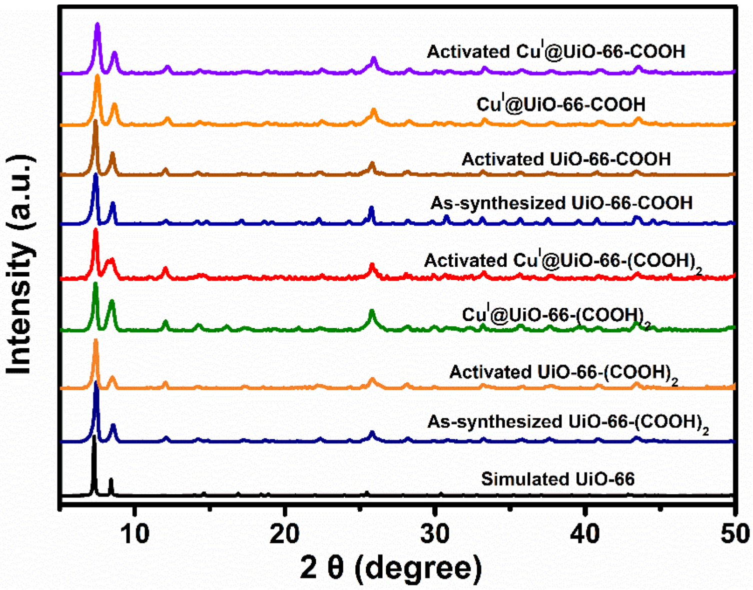

Figure S1. The PXRD patterns for the synthesized and activated UiO-66 series MOF materials along with the simulated XRD pattern of UiO-66 (black) derived from the simulated crystal structure.

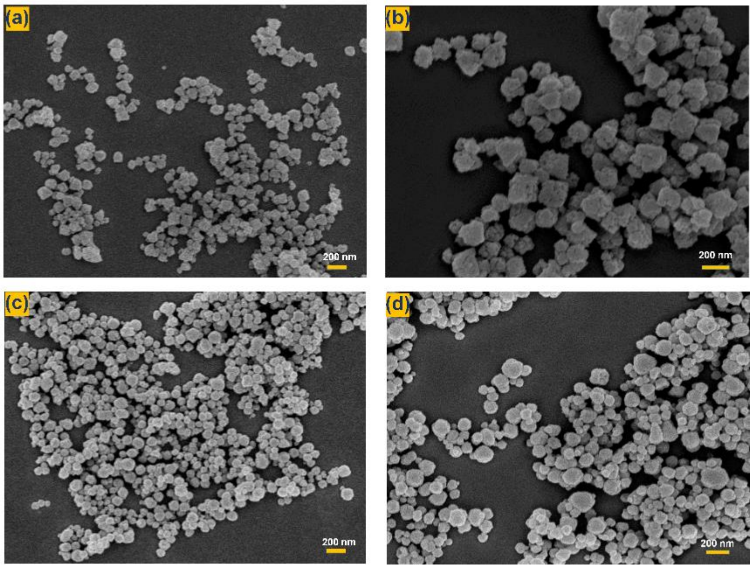

Figure S2. SEM images of UiO-66-COOH (a), Cu^{I}@UiO-66-COOH (b), UiO-66-$(COOH)_2$ (c) and Cu^{I}@UiO-66-$(COOH)_2$, which feature particle sizes of around 100~200 nm.

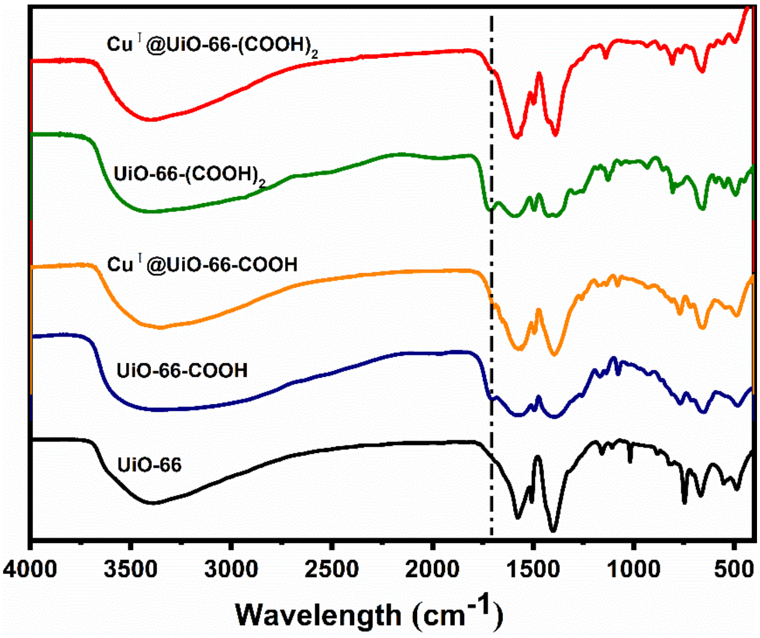

Figure S3. FT-IR spectra of UiO-66 series MOFs. One hand, the strong bands at 1715.7 cm^{-1} attributed to C=O stretching vibration of uncoordinated -COOH groups were observed in UiO-66-COOH and $-(COOH)_2$, but not found in UiO-66. On the other hand, such characteristic peaks were almost disappeared in both Cu^I@UiO-66-$(COOH)_2$ and Cu^I@UiO-66-COOH, further confirming it is the exposed carboxyl groups that play the coordination role in chelating Cu(I) ions.

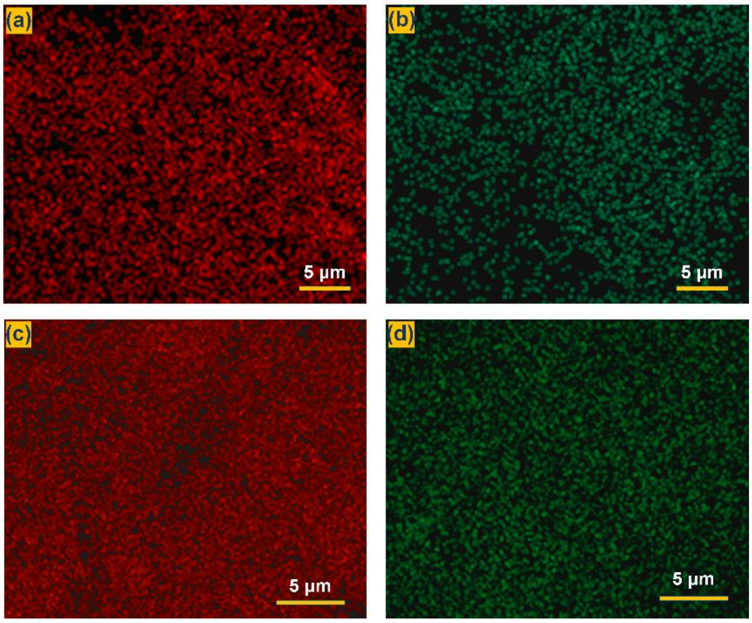

Figure S4. Element maps for oxygenium (O) of Cu^{I}@UiO-66-COOH (a) and Cu^{I}@UiO-66-$(COOH)_2$ (c); Element maps for Cuprum (Cu) of Cu^{I}@UiO-66-COOH (b) and Cu^{I}@UiO-66-$(COOH)_2$ (d).

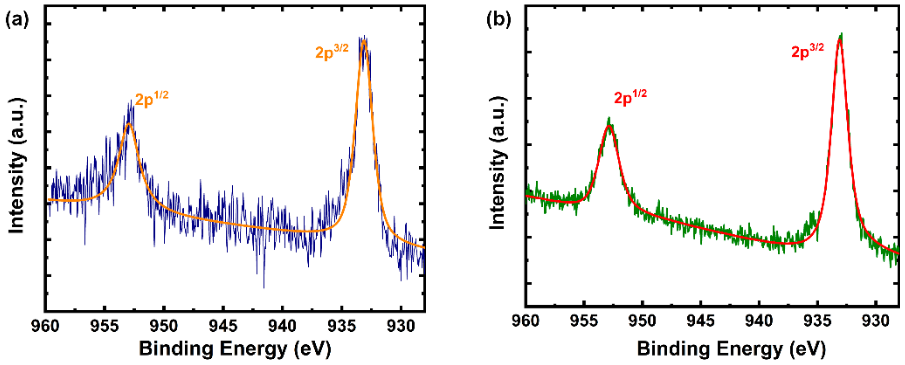

Figure S5. (a) XPS for Cu(I) sites of Cu^I@UiO-66-COOH after etching of surface; (b) XPS for Cu(I) sites of Cu^I@UiO-66-$(COOH)_2$ after etching of surface. The results show that a characteristic signal from copper(I) at binding energies of 952.9ev and 933.1ev is corresponding to the peaks of Cu $2p^{1/2}$ and $2p^{3/2}$ respectively.

(a)

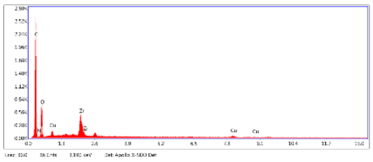

Element	Weight %	Atom %
C K	58.0	66.94
N K	5.80	5.69
O K	30.95	26.58
Zr L	3.60	0.54
Cu K	1.15	0.25
Total	100.00	

(b)

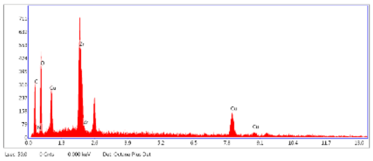

Element	Weight %	Atom %
C K	30.74	48.42
N K	5.32	7.19
O K	30.63	36.22
Zr L	19.34	4.01
Cu K	13.96	4.16
Total	100.00	

Figure S6. (a) Element Analysis for Cu^{I}@UiO-66-COOH by EDS; (b) Element Analysis for Cu^{I}@UiO-66-$(COOH)_2$ by EDS.

Table S1. The ICP-MS results for Cu^{I}@UiO-66-COOH and Cu^{I}@UiO-66-$(COOH)_2$.

Samples	Zr^{4+} (ppm)	Cu^{+} (ppm)	Molar Ratio (Zr^{4+}: Cu^{+})
Cu^{I}@UiO-66-COOH	5.69	1.97	1:0.47
Cu^{I}@UiO-66-$(COOH)_2$	4.87	3.56	1:1.05

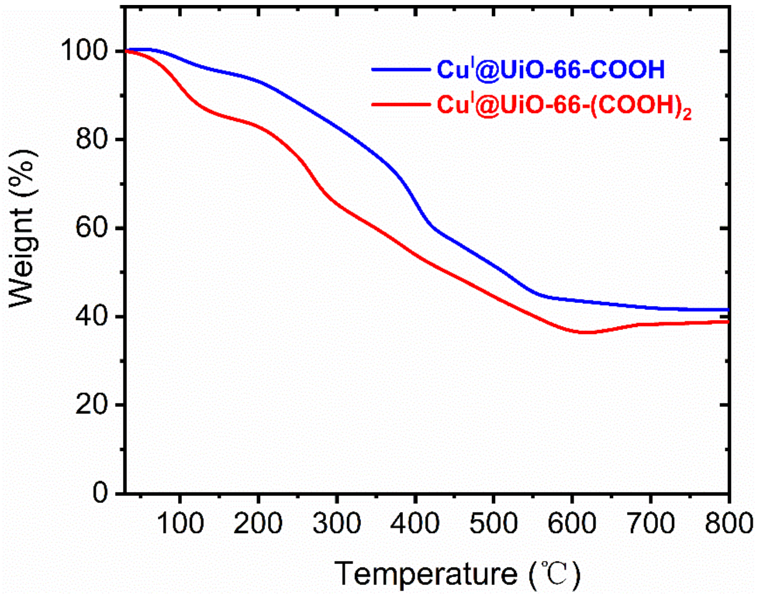

Figure S7. Thermogravimetric analysis (TGA) curves of Cu^{I}@UiO-66-COOH and Cu^{I}@UiO-66-$(COOH)_2$.

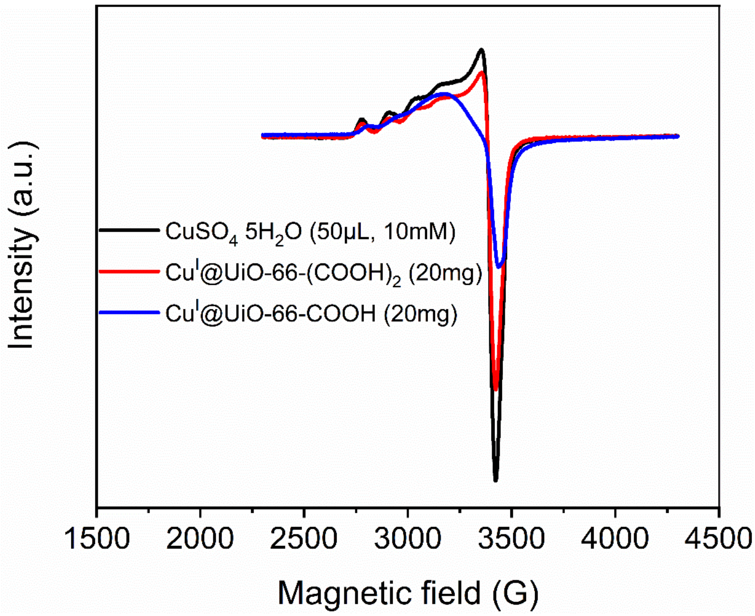

Figure S8. The ESR spectra of Cu^I@UiO-66-COOH and Cu^I@UiO-66-$(COOH)_2$.The corresponding results of n(Cu^I): n(Cu^{II}) are 8.3 :1 and 11.6:1 for Cu^I@UiO-66-COOH and Cu^I@UiO-66-$(COOH)_2$ respectively. The existing of Cu^{II} ions is because the inevitable oxidation of Cu^I during the sample fabrication even in the glove box.

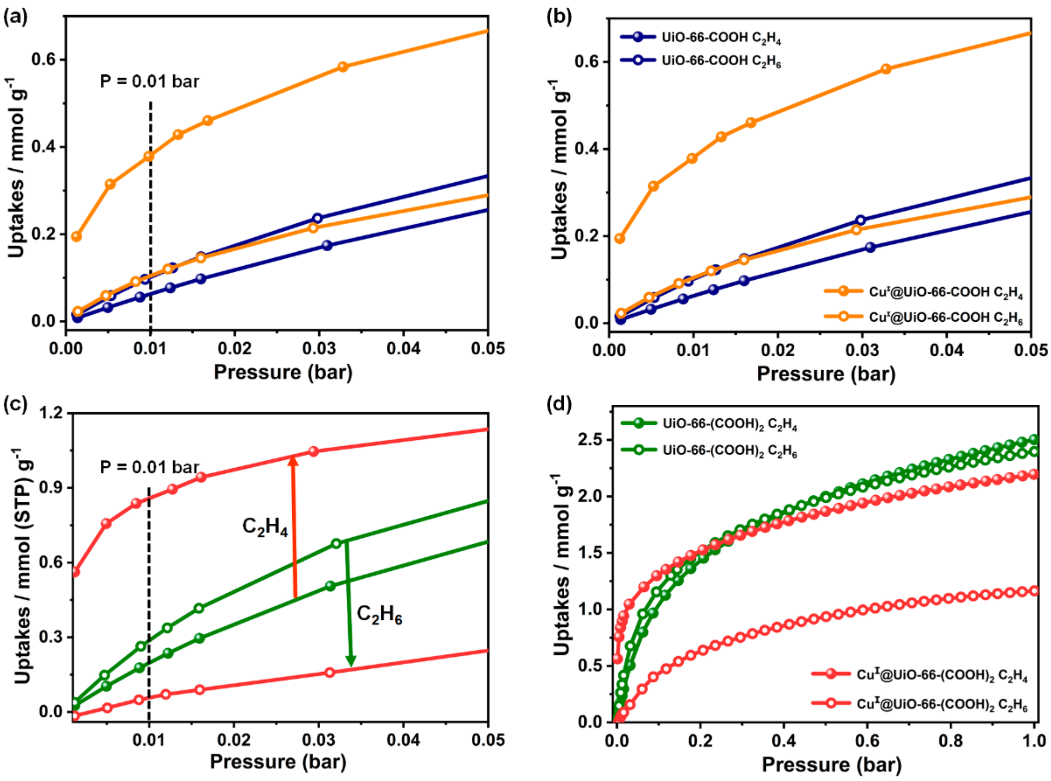

Figure S9. (a) Experimental C_2H_4 and C_2H_6 adsorption isothermals of UiO-66-COOH and Cu^I@UiO-66-COOH in the region of 0-0.05 bar at 273 K. (b) Single-component adsorption isotherms for C_2H_4 and C_2H_6 of UiO-66-COOH and Cu^I@UiO-66-COOH at 273 K. (c) Experimental C_2H_4 and C_2H_6 adsorption isothermals of UiO-66-$(COOH)_2$ and Cu^I@UiO-66-$(COOH)_2$ in the region of 0-0.05 bar at 273 K. (d) Single-component adsorption isotherms for C_2H_4 and C_2H_6 of UiO-66-$(COOH)_2$ and Cu^I@UiO-66-$(COOH)_2$ at 273 K.

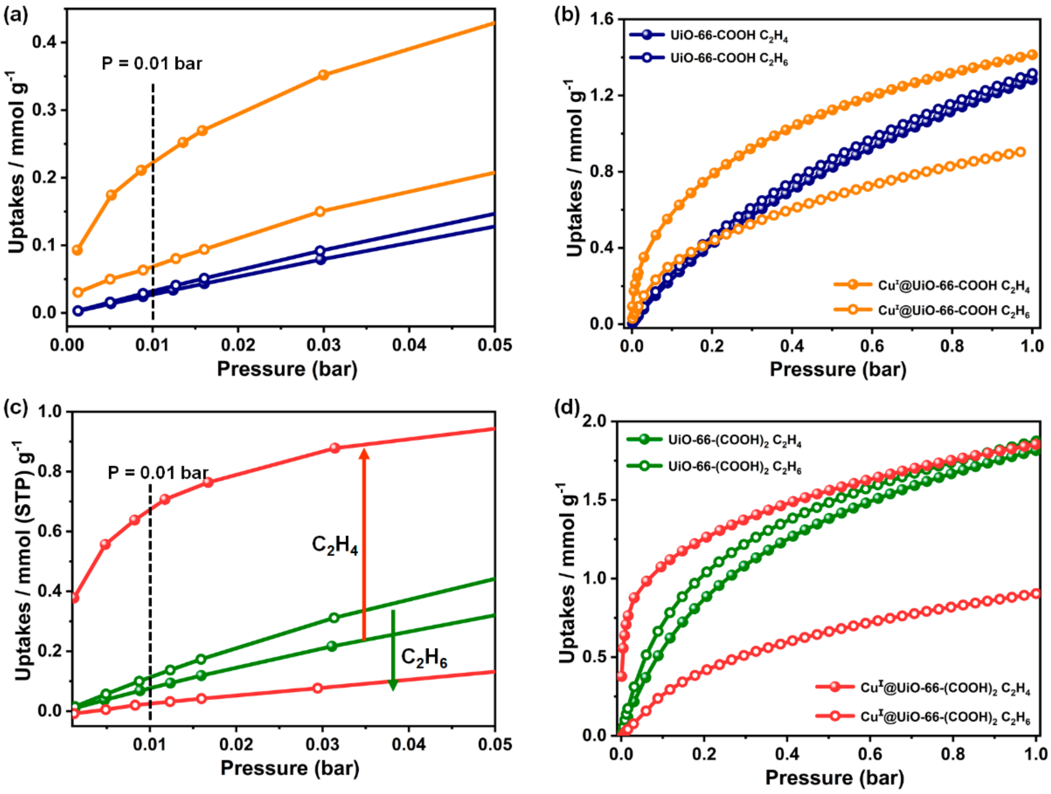

Figure S10. (a) Experimental C_2H_4 and C_2H_6 adsorption isothermals of UiO-66-COOH and Cu^I@UiO-66-COOH in the region of 0-0.05 bar at 298 K. (b) Single-component adsorption isotherms for C_2H_4 and C_2H_6 of UiO-66-COOH and Cu^I@UiO-66-COOH at 298 K. (c) Experimental C_2H_4 and C_2H_6 adsorption isothermals of UiO-66-$(COOH)_2$ and Cu^I@UiO-66-$(COOH)_2$ in the region of 0-0.05 bar at 298 K. (d) Single-component adsorption isotherms for C_2H_4 and C_2H_6 of UiO-66-$(COOH)_2$ and Cu^I@UiO-66-$(COOH)_2$ at 298 K.

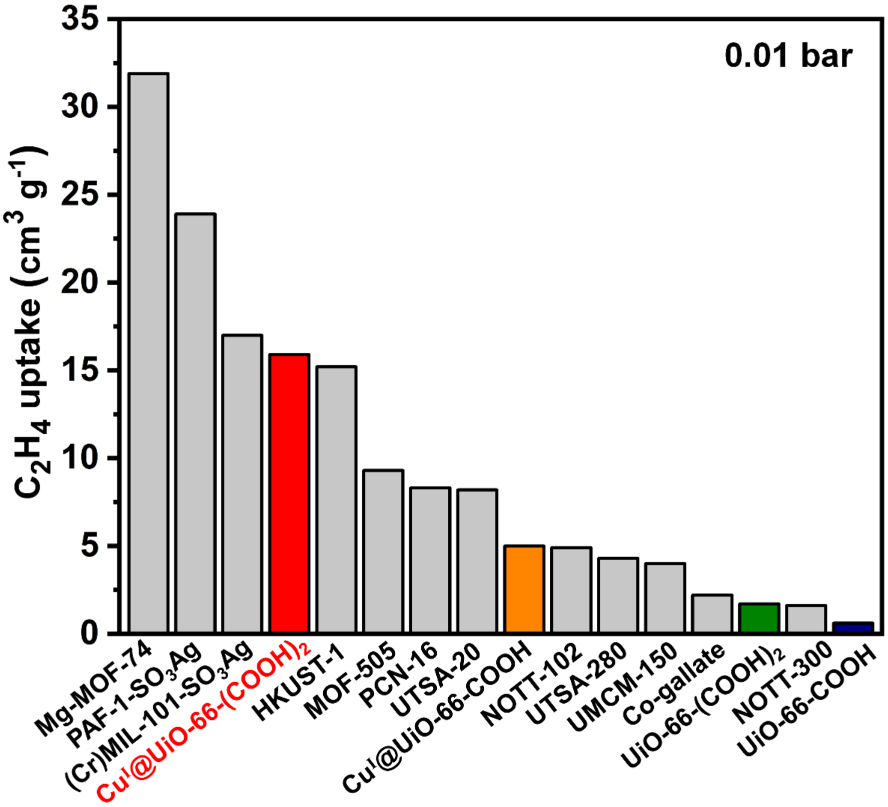

Figure S11. Comparison of C_2H_4 uptake capacity for UiO-66-type MOFs and other top-performing materials at 0.01 bar and room temperature.

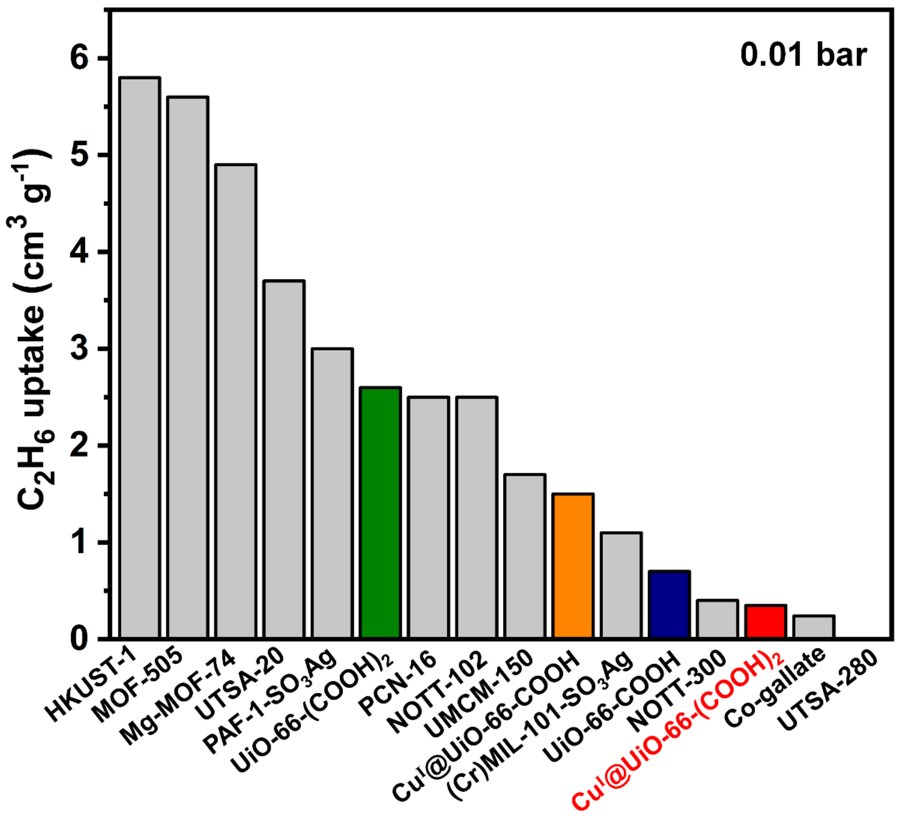

Figure S12. Comparison of C_2H_6 uptake capacity for UiO-66-type MOFs and other top-performing materials at 0.01 bar and room temperature.

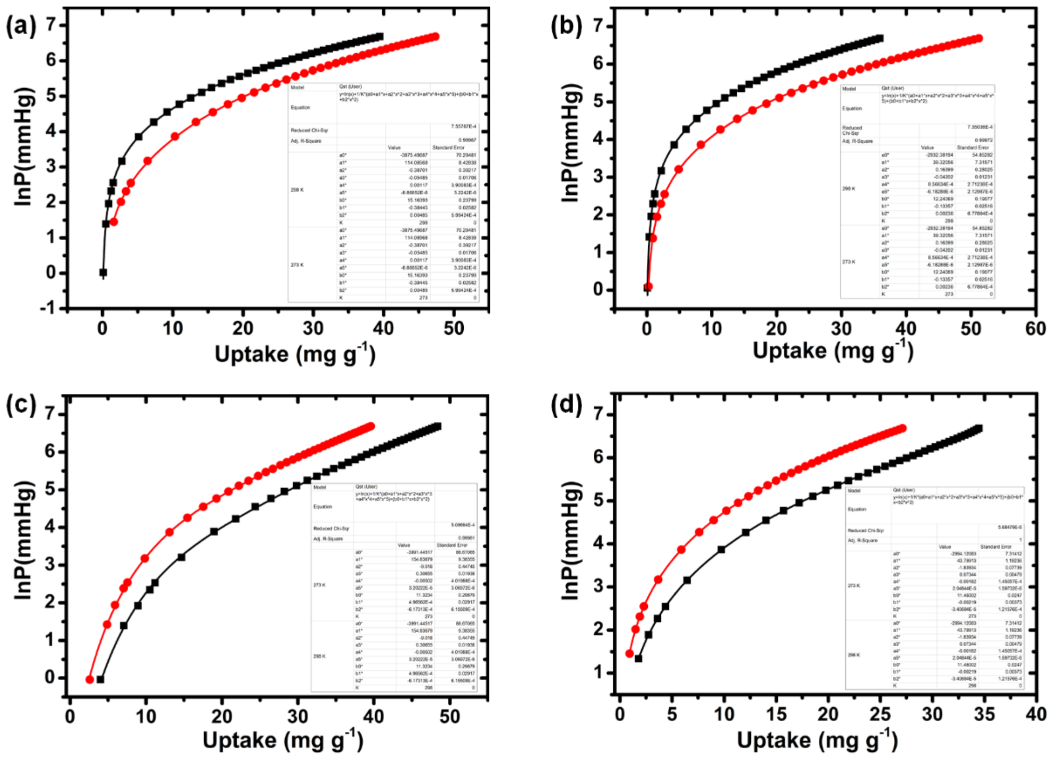

Figure S13. The fitting by the virial equation of C_2H_4 and C_2H_6 in UiO-66-COOH and Cu^{I}@UiO-66-COOH.

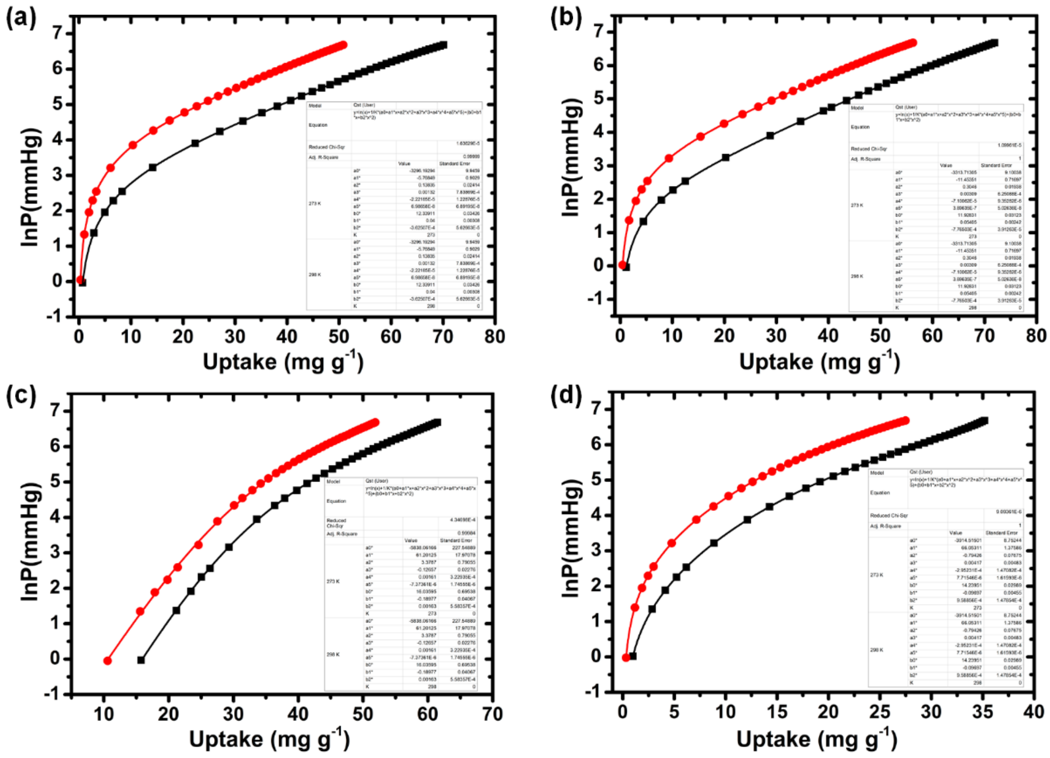

Figure S14. The fitting by the virial equation of C_2H_4 and C_2H_6 in UiO-66-(COOH)$_2$ and Cu^I@UiO-66-(COOH)$_2$.

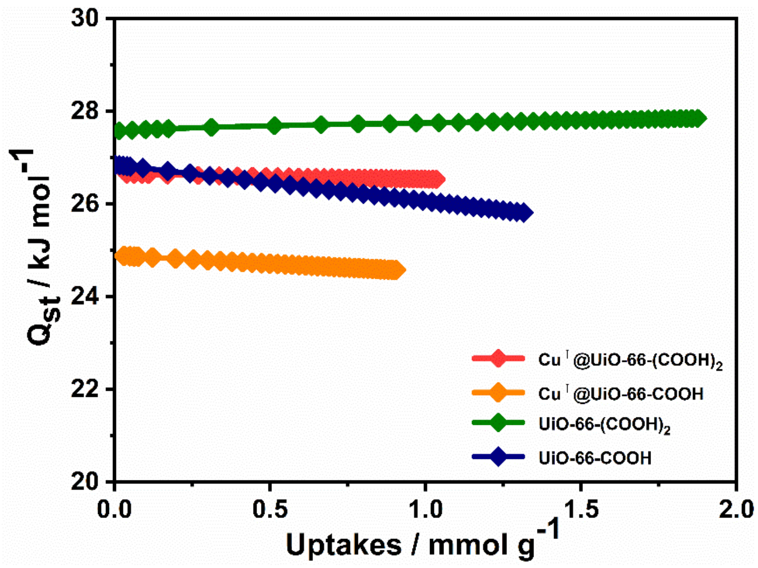

Figure S15. The isosteric heat (Q_{st}) of C_2H_6 in UiO-66-COOH, UiO-66-$(COOH)_2$ Cu^I@UiO-66-COOH and Cu^I@UiO-66-$(COOH)_2$.

Table S2. Langmuir-Freundlich parameter fits for C_2H_6 at 273 K in these four UiO-66 series MOFs.

	*N*max (mmol/g)	*b* (kPa^{-1})	1/n
UiO-66-COOH	4.49528	0.02554	0.68978
Cu^{I}@UiO-66-COOH	2.04541	0.05375	0.69262
UiO-66-$(COOH)_2$	3.36843	0.10839	0.66877
Cu^{I}@UiO-66-$(COOH)_2$	1.60274	0.04548	0.88138

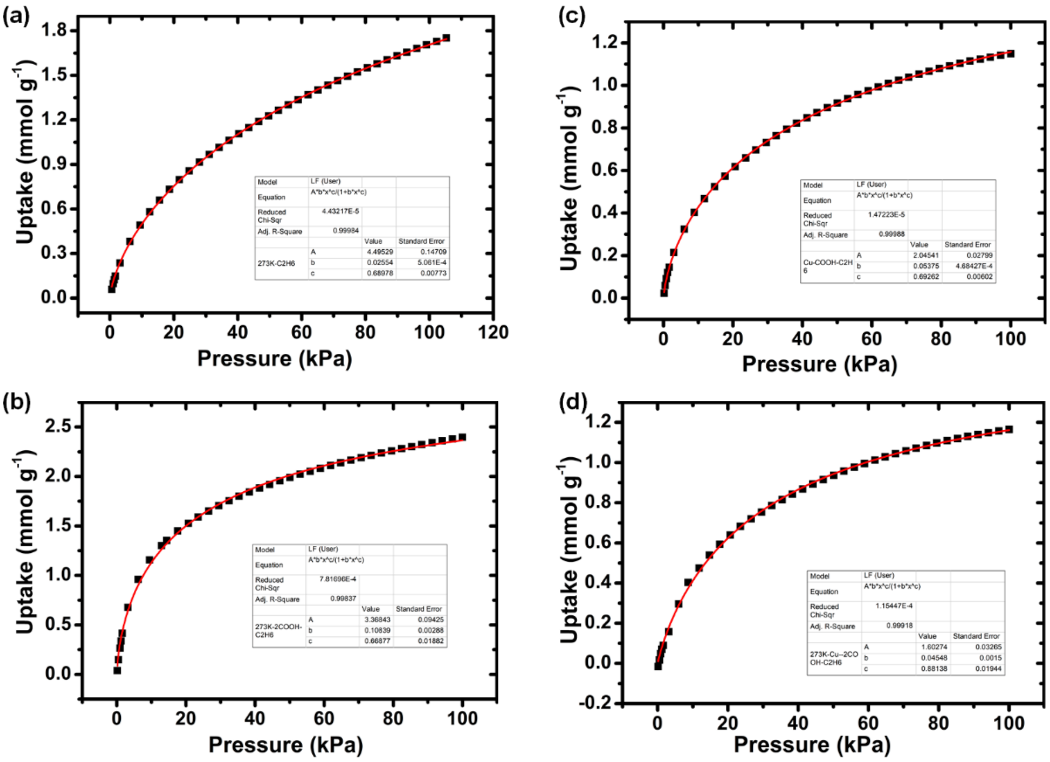

Figure S16. Langmuir-Freundlich fit to the adsorption data of C_2H_6 at 273 K in UiO-66-COOH (a), UiO-66-$(COOH)_2$ (b), Cu^I@UiO-66-COOH (c) and Cu^I@UiO-66-$(COOH)_2$ (d).

Table S3. Langmuir-Freundlich parameter fits for C_2H_4 at 273 K in these four UiO-66-type MOFs.

	N_1max (mmol/g)	b_1 (kPa^{-1})	$1/n_1$	N_2max (mmol/g)	b_2 (kPa^{-1})	$1/n_2$
UiO-66 -COOH	4.49172	0.01615	0.80418	/	/	/
Cu^I@UiO -66-COOH	7.30984	0.05565	0.37875	/	/	/
UiO-66 -$(COOH)_2$	3.69754	0.06913	0.73128	/	/	/
Cu^I@UiO-66 -$(COOH)_2$	2.8289	0.0248	0.69977	1.2014	1.94716	0.40094

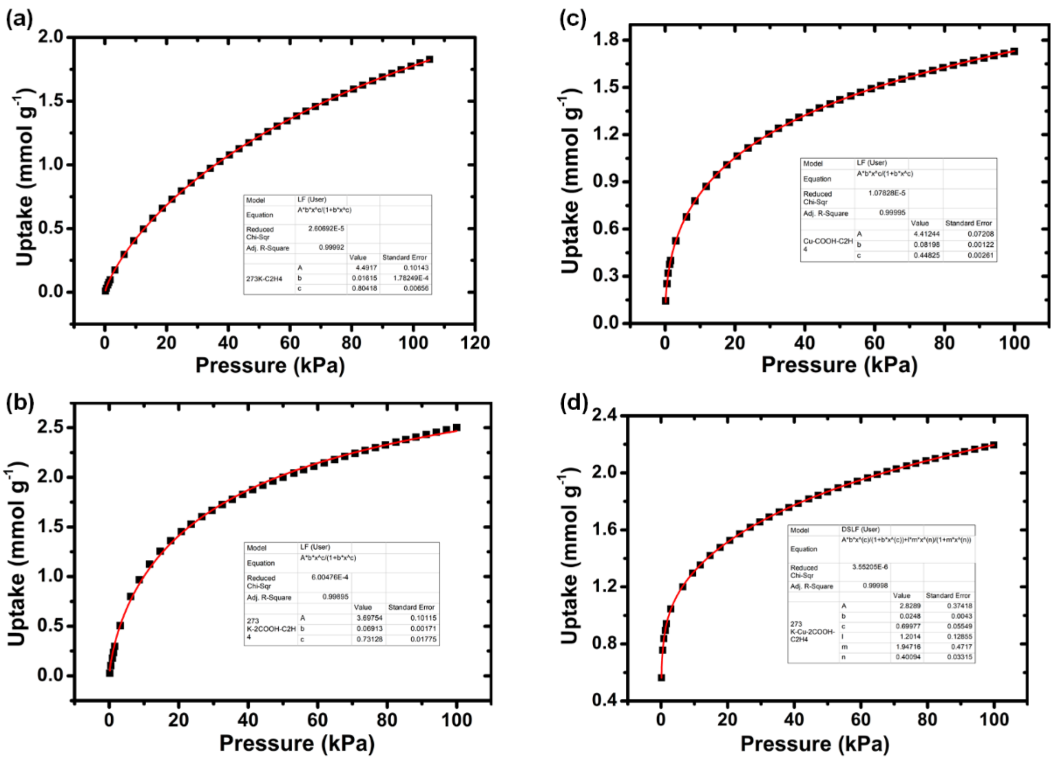

Figure S17. Langmuir-Freundlich fit to the adsorption data of C_2H_4 at 273 K in UiO-66-COOH (a), UiO-66-$(COOH)_2$ (b), Cu^I@UiO-66-COOH (c) and Cu^I@UiO-66-$(COOH)_2$ (d).

Table S4. Langmuir-Freundlich parameter fits for C_2H_6 at 298 K in these four UiO-66-type MOFs.

	N_{max} (mmol/g)	b (kPa^{-1})	1/n
UiO-66-COOH	3.45503	0.01078	0.86771
Cu^{I}@UiO-66-COOH	2.78925	0.03014	0.60317
UiO-66-$(COOH)_2$	2.70791	0.05456	0.79817
Cu^{I}@UiO-66-$(COOH)_2$	1.47707	0.02687	0.87713

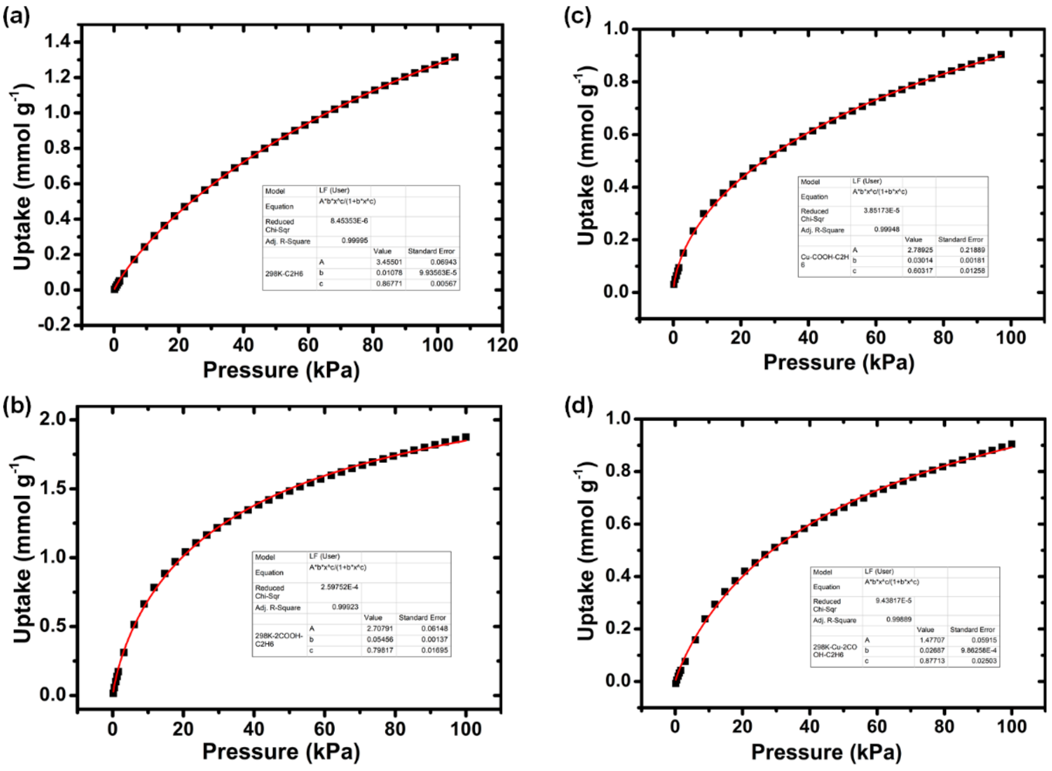

Figure S18. Langmuir-Freundlich fit to the adsorption data of C_2H_6 at 298 K in UiO-66-COOH (a), UiO-66-$(COOH)_2$ (b), Cu^I@UiO-66-COOH (c) and Cu^I@UiO-66-$(COOH)_2$ (d).

Table S5. Langmuir-Freundlich parameter fits for C_2H_4 at 298 K in these four UiO-66-type MOFs.

	N_1max (mmol/g)	b_1 (kPa^{-1})	1/n_1	N_2max (mmol/g)	b_2 (kPa^{-1})	1/n_2
UiO-66 -COOH	4.49172	0.01615	0.80418	/	/	/
Cu^I@UiO -66-COOH	7.30984	0.05565	0.37875	/	/	/
UiO-66 -$(COOH)_2$	3.69754	0.06913	0.73128	/	/	/
Cu^I@UiO-66 -$(COOH)_2$	2.8289	0.0248	0.69977	1.2014	1.94716	0.40094

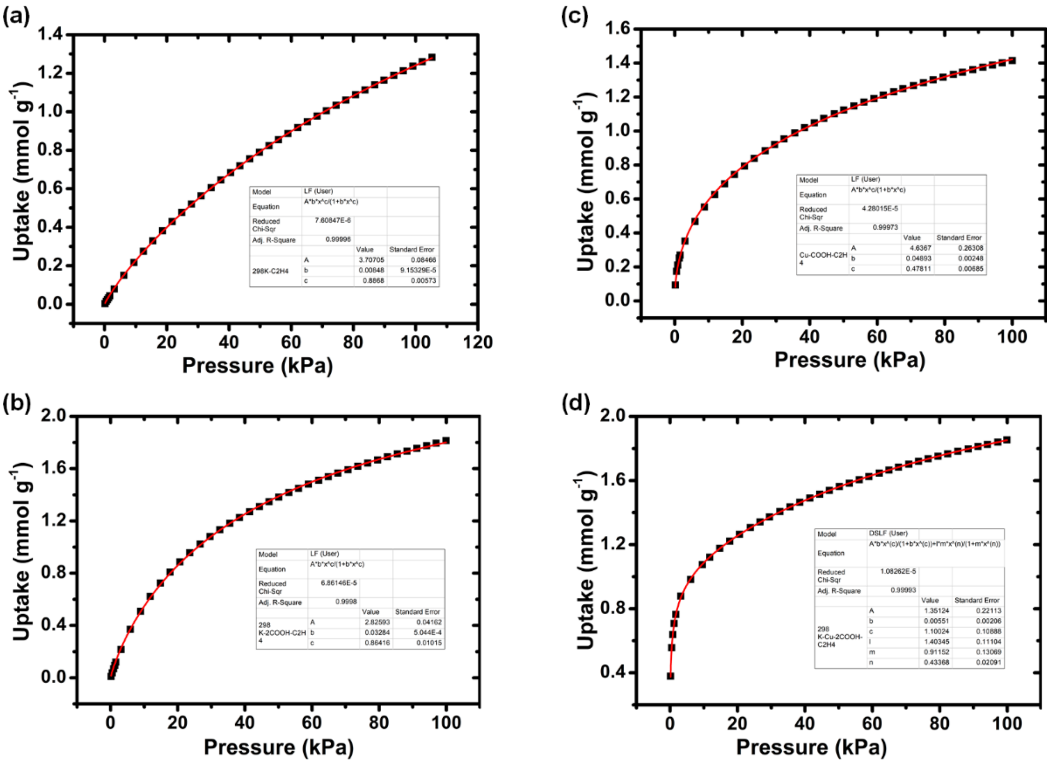

Figure S19. Langmuir-Freundlich fit to the adsorption data of C_2H_4 at 298 K in UiO-66-COOH (a), UiO-66-$(COOH)_2$ (b), Cu^I@UiO-66-COOH (c) and Cu^I@UiO-66-$(COOH)_2$ (d).

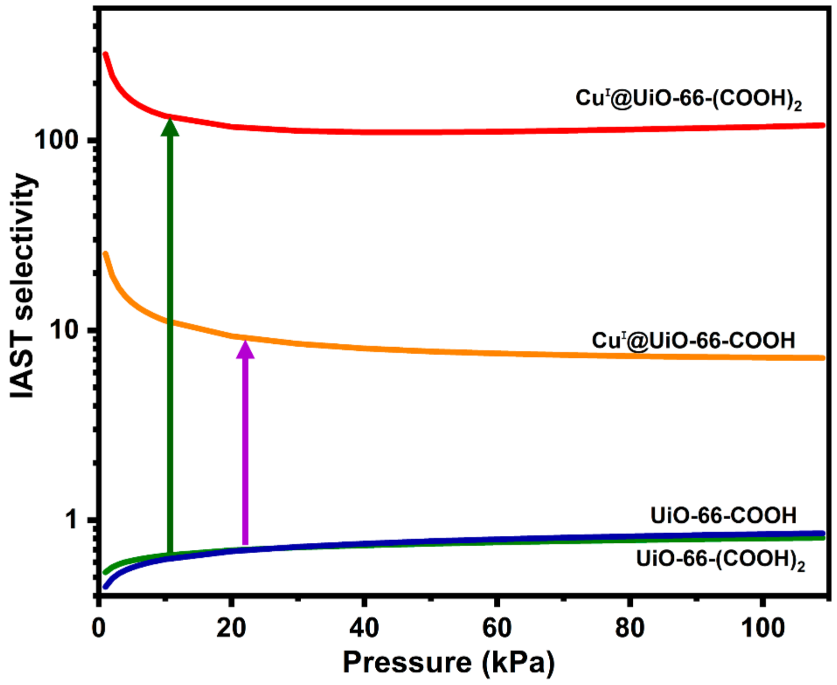

Figure S20. IAST calculations of activated UiO-66 series MOFs for the C_2H_4/ C_2H_6 separation at 273 K.

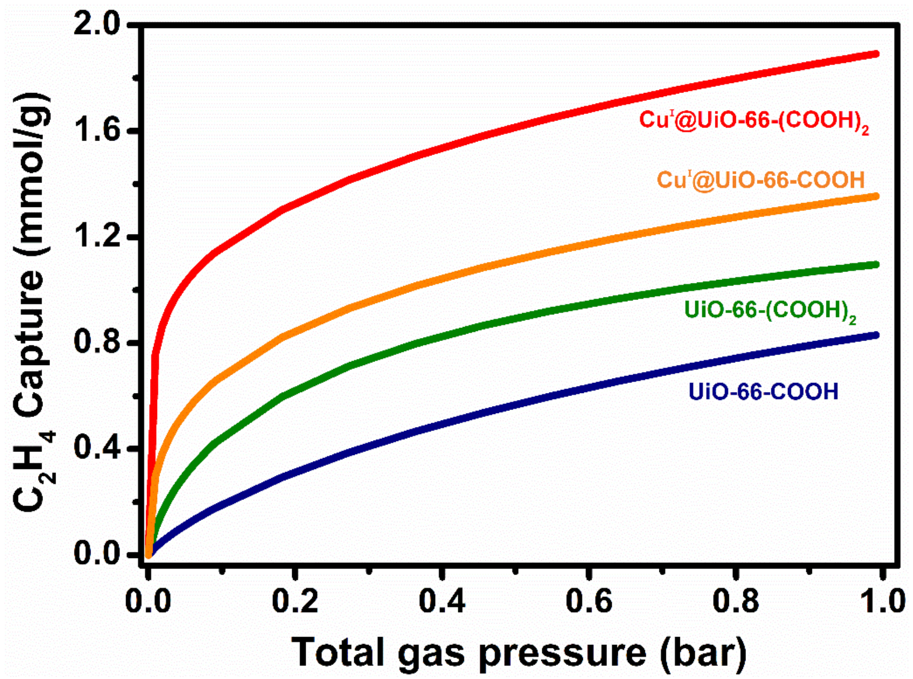

Figure S21. IAST calculated C_2H_4 uptake of UiO-66-type MOFs from C_2H_4/C_2H_6 (50:50; v:v) gas mixtures at 273 K. Cu^I@UiO-66-$(COOH)_2$ adsorbs C_2H_4 rapidly and exhibits the highest C_2H_4 uptake (1.89 mmol/g) among the four UiO-66-type MOFs.

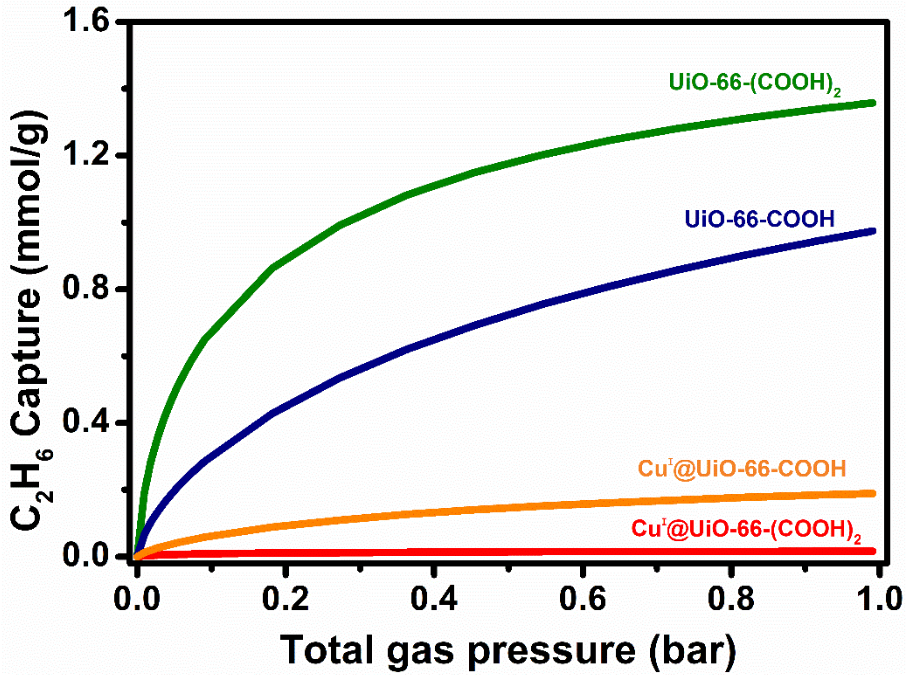

Figure S22. IAST calculated C_2H_6 uptake of UiO-66-type MOFs from C_2H_4/C_2H_6 (50:50; v:v) gas mixtures at 273 K. Cu^I@UiO-66-$(COOH)_2$ almost completely exclude C_2H_6 from the C_2H_4/C_2H_6 mixture as the result of selective size exclusion.

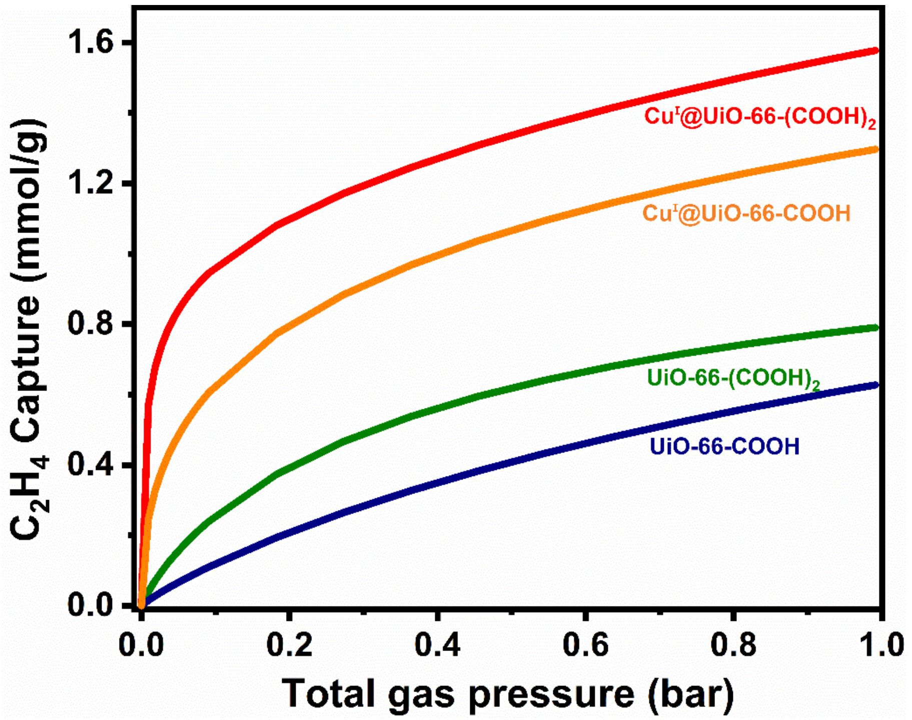

Figure S23. IAST calculated C_2H_4 uptake of UiO-66-type MOFs from C_2H_4/C_2H_6 (50:50; v:v) gas mixtures at 298 K. Cu^I@UiO-66-$(COOH)_2$ adsorbs C_2H_4 rapidly and exhibits the highest C_2H_4 uptake (1.58 mmol/g) among the four UiO-66-type MOFs.

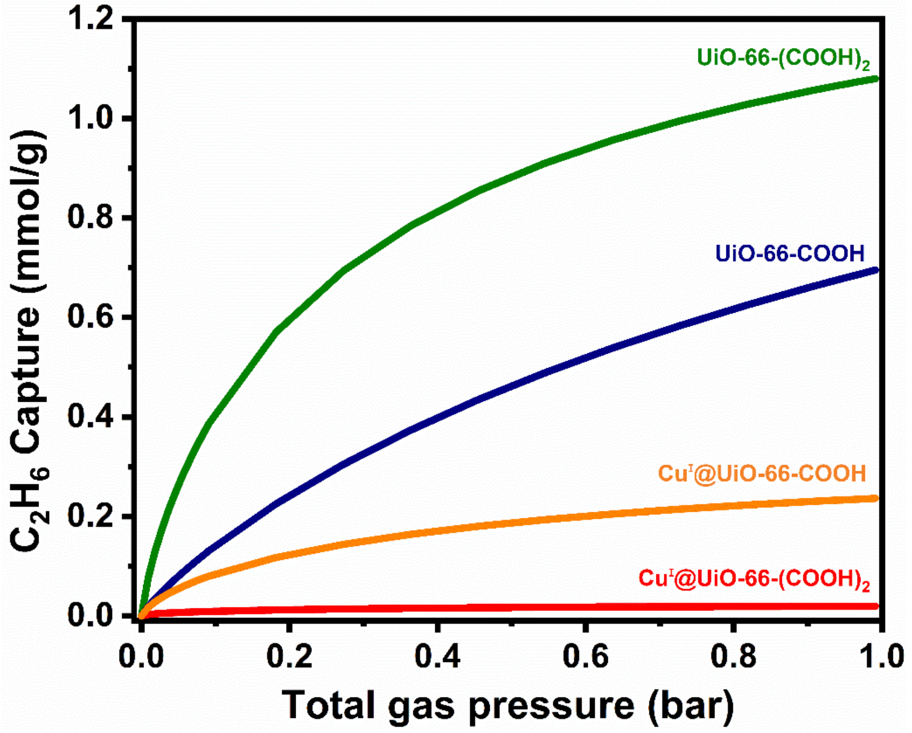

Figure S24. IAST calculated C_2H_6 uptake of UiO-66-type MOFs from C_2H_4/C_2H_6 (50:50; v:v) gas mixtures at 298 K. Cu^I@UiO-66-(COOH)$_2$ almost completely exclude C_2H_6 from the C_2H_4/C_2H_6 mixture as the result of selective size exclusion.

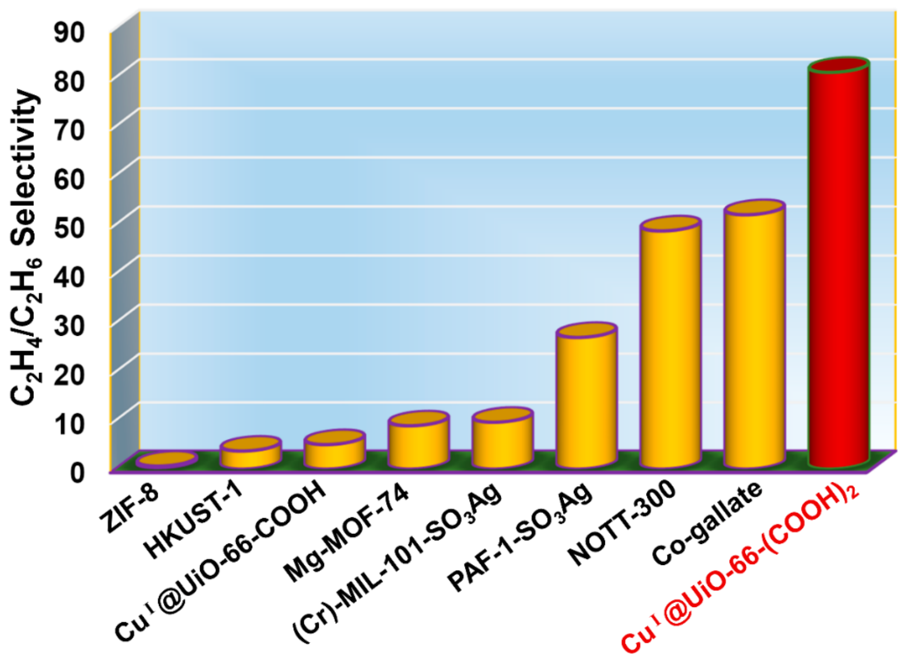

Figure S25. Comparison of the selectivity of C_2H_4/C_2H_6 among representative porous materials.

Table S6. Summary of the adsorption uptakes, uptake ratio and heat of adsorption data for C_2H_4 in promising ethylene sorbents.

Porous Materials	Surface area (m^2/g, BET)	C_2H_4 uptake at 0.5 bar (mmol/g)	C_2H_6 uptake at 0.5 bar (mmol/g)	Uptake ratio of C_2H_4/C_2H_6[a]	Qst of C_2H_4 (kJ/mol)[b]
NaX[1]	525	2.65	1.97	1.35	38.10
Zeolite-5Å[2]	457-600	2.20	1.46	1.51	37
NaETS-10[3]	289	1.62	1.27	1.28	/
ITQ-55[4]	/	1.17	0.76	1.54	/
Co-MOF-74[5]	1341	5.57	4.17	1.34	43.6
Fe-MOF-74[6]	1350	5.87	4.02	1.46	47.5
Fe_2(m-dobdc)[7]	1295	6.39	5.07	1.26	55
NOTT-300[8]	1370	3.20	0.56	5.71	16
PAF-1-SO_3Ag[9]	783	3.33	1.97	1.69	106
(Cr)-MIL-101-SO_3Ag[10,11]	1374	2.41	1.21	1.99	63
UiO-66-$(COO)_2$	622	1.38	1.48	0.93	27.4
Cu^I@UiO-66-$(COOH)_2$	320	1.56	0.66	2.36	48.5

[a] Uptake ratio of C_2H_4/C_2H_6 at 0.05 bar;

[b] Q_{st} values at zero coverage.

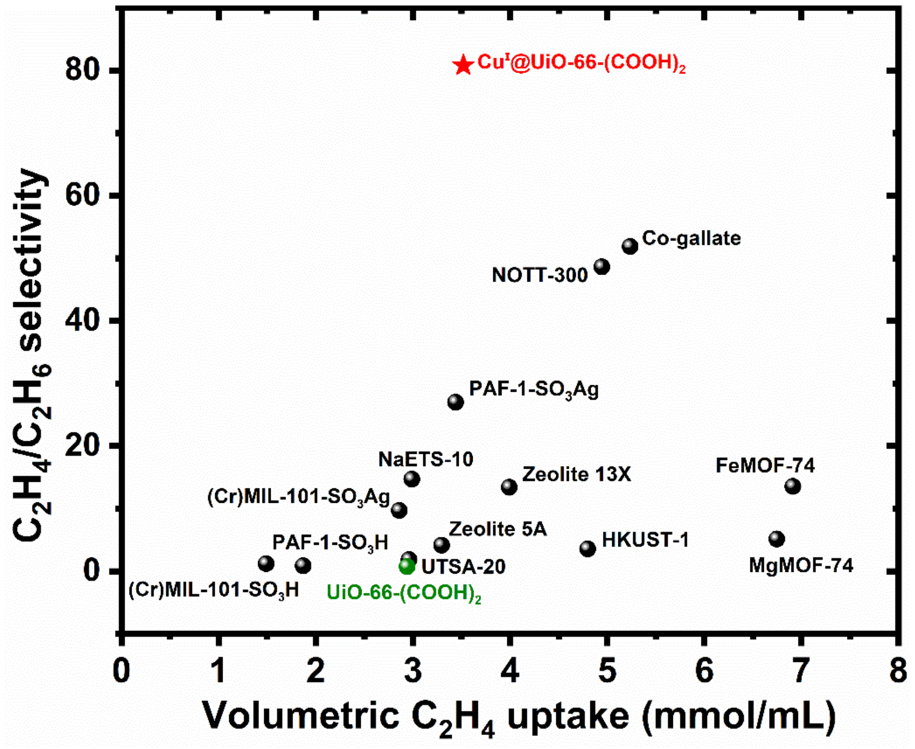

Figure S26. Comparison of C_2H_4/C_2H_6 adsorption selectivity and C_2H_4 volumetric uptake at 1.0 bar in promising porous materials.

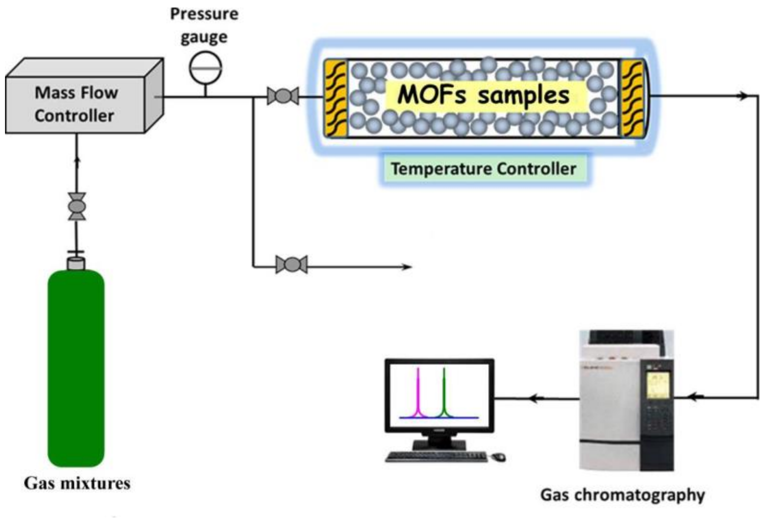

Figure S27. The procedures for the breakthrough test of activated Cu^I@UiO-66-$(COOH)_2$ for C_2H_4/C_2H_6 (v : v, 50 : 50) at room temperature.

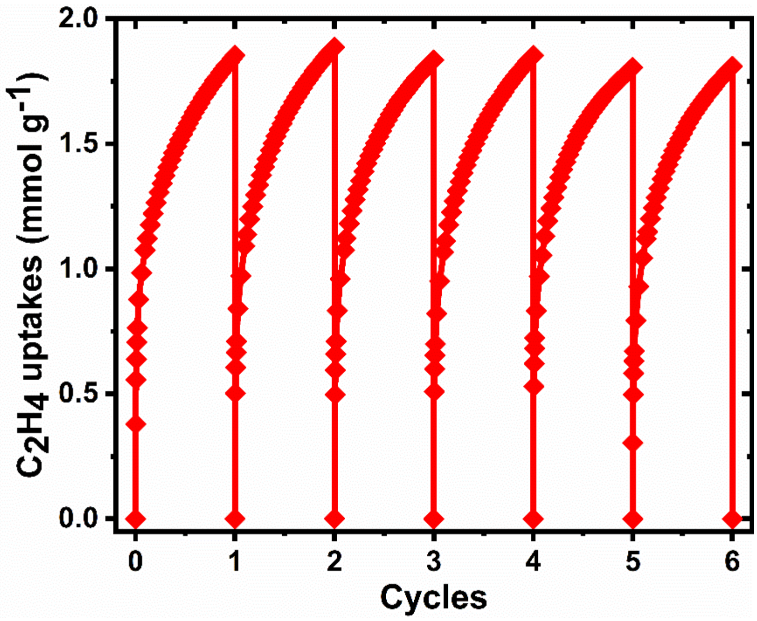

Figure S28. Cycles of C_2H_4 adsorption for Cu^I@UiO-66-$(COOH)_2$ at 298 K, performing repeated test without any treatment in the adsorption tube.

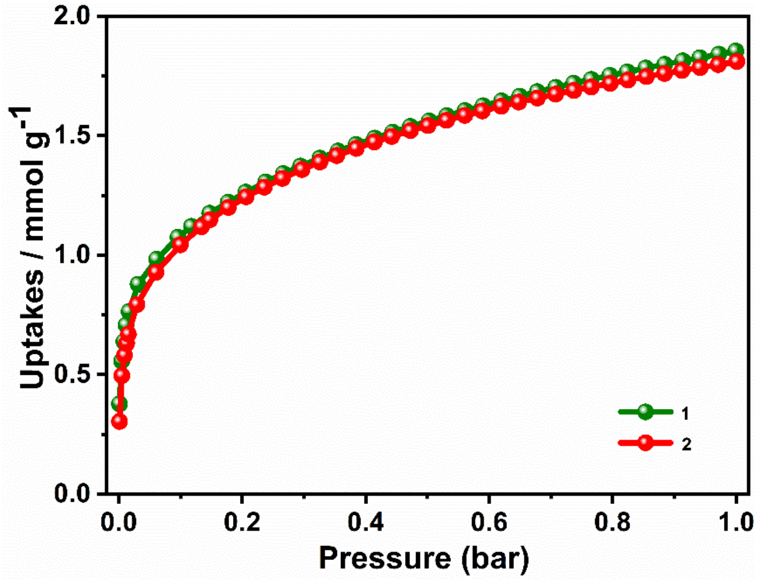

Figure S29. The C_2H_4 adsorption isotherms at 298 K of the activated Cu^I@UiO-66-$(COOH)_2$ (**1**) and the reactivation Cu^I@UiO-66-$(COOH)_2$ (**2**) after soaking in the deionized water with the absence of oxygen for 1 day.

Reference

[1] S. H. Hyun, R. P. Danner, *J. Chem. Eng. Data* **1982**, *27*, 196.

[2] M. Mofarahi, S. M. Salehi, *Adsorption* **2012**, *19*, 101.

[3] A. Anson, Y. Wang, C. C. H. Lin, T. M. Kuznicki, S. M. Kuznicki, *Chem. Eng. Sci.* **2008**, *63*, 4171.

[4] Bereciartua, P. J. et al. *Science* **2017**, *358*, 1068.

[5] S. J. Geier, J. A. Mason, E. D. Bloch, W. L. Queen, M. R. Hudson, C. M. Brown, J. R. Long, Chem. Sci. 2013, 4, 2054.

[6] E. D. Bloch, W. L. Queen, R. Krishna, J. M. Zadrozny, C. M. Brown, J. R. Long, *Science* **2012**, *335*, 1606.

[7] Bachman, J. E., Kapelewski, M. T., Reed, D. A., Gonzalez, M. I. & Long, J. R. *J. Am. Chem. Soc.*, **2017**,*139*, 15363.

[8] S. Yang, A. J. Ramirez-Cuesta, R. Newby, V. Garcia-Sakai, P. Manuel, S. K. Callear, S. I. Campbell, C. C. Tang, M. Schroder, *Nat. Chem.* **2014**, *7*, 121.

[9] B. Li, Y. Zhang, R. Krishna, K. Yao, Y. Han, Z. Wu, D. Ma, Z. Shi, T. Pham, B. Space, J. Liu, P. K. Thallapally, J. Liu, M. Chrzanowski, S. Ma, J. *Am. Chem. Soc.* **2014**, *136*, 8654.

[10] G. Chang, M. Huang, Y. Su, H. Xing, B. Su, Z. Zhang, Q. Yang, Y. Yang, Q. Ren, Z. Bao, B. Chen, *Chem. Commun.* **2015**, *51*, 2859.

[11] Y. Zhang, B. Li, R. Krishna, Z. Wu, D. Ma, Z. Shi, T. Pham, K. Forrest, B. Space, S. Ma, *Chem. Commun.* **2015**, *51*, 2714.
